# Incidence of reported cases of euthanasia adjusted for demographic composition: a study of ten years of Belgian administrative data (2014–2023)

**DOI:** 10.1186/s12889-025-24839-x

**Published:** 2025-11-07

**Authors:** Natasia Hamarat, Jacques Wels

**Affiliations:** 1 Université libre de Bruxelles, Health & Society Research Unit, Av. Jeanne 44 - CP 124, Brussels, 1050 Belgium; 2 University College London, Unit for Lifelong Health and Ageing, London, United Kingdom

**Keywords:** Euthanasia, Assisted dying, Belgium, Demographics, Poisson regression, Death, Psychiatric disorders, Cancers

## Abstract

**Background:**

Cases of reported euthanasia or assisted dying have increased in all countries that provide such legislation. In Belgium, the number of annual reported cases rose from 1,928 in 2014 to 3,423 in 2023. However, no study has addressed how this change reflects demographic composition. Using Belgian administrative data, the study shows actual trends and how population composition explains variations across sub-groups including age, gender, region and reason for euthanasia.

**Methods:**

We use complete micro-data on all cases of euthanasia reported between 2014 and 2023 (*N* = 24,840) gathered by the Belgian Federal Commission for the Control and Evaluation of Euthanasia (FCCEE). We apply Poisson regression controlling for time and use interaction terms to address time change over subgroups and provide Incidence Rate Ratios (IRR). We compare net estimates with a modelling weighting for population demographics generated from the Belgian Office for Statistics data.

**Results:**

Data show an IRR of euthanasia of 1.06 (95%CI = 1.056;1.066) – i.e., an increase of 6% per year. Weighted for population characteristics, the IRR is 1.04 (95%CI = 1.039;1.049). Demographic composition explains such a difference, not demographic change. Unweighted data show higher incidence amongst female [male = 0.934 (95%CI = 0.911;0.958)] but the trend is reversed when weighting for demographics [male = 1.076 (95%CI = 1.046;1.105)]. Gender differences in reasons for euthanasia exist with cancers and psychiatric disorders more often observed in male and female respectively. Euthanasia is more common in the Flanders [3.058 (95%CI = 2.949;3.171)] and the demographic adjustment does not fully reduce the regional divide.

**Conclusions:**

Analysis on euthanasia and assisted dying should consider population demographics when addressing incidence amongst populations to better capture age, regional and gender differences.

**Supplementary Information:**

The online version contains supplementary material available at 10.1186/s12889-025-24839-x.

## Background

 The number of reported cases of euthanasia has almost constantly increased in countries that have enacted regulations on euthanasia or assisted dying. In the Netherlands, 1,933 cases were reported in 2005 against 6,361 in 2019 [1] and 9,068 in 2023 [2]. As the percentage of all death in the population, euthanasia accounted for 1.9% in 1990, 4.4% in 2017 and 5.4% in 2023 [2]. In Switzerland, cohort data from 1991 to 2008 have shown that the number of older women requesting assisted dying has tripled whilst it has doubled for men [3]. This comes together with a gradual positive change in attitudes towards euthanasia that is observed over time [4] but varies by country [5] and an increasing number of countries implementing or debating some kind of assisted dying policies [6].

In Belgium, the number of reported cases of euthanasia was 235 in 2003 and 1,807 in 2013 [7]. They were 2,700 in 2021 [8]. In 20 years, the figure was multiplied by seven; euthanasia was estimated to represent 2.4% of all deaths reported in Belgium in 2021 [8]. Belgium legalized euthanasia in May 2002 [9], becoming the second country after the Netherlands to do so. The Belgian Euthanasia Act allows legally competent adults experiencing “constant and unbearable physical or mental suffering that cannot be alleviated” due to a serious and incurable medical condition to request euthanasia. The request must be voluntary, deliberate, and repeated, and it must be made in writing. One or two independent physicians - either psychiatrist or specialist of the pathology - who are not affiliated with the attending physician or the patient and are knowledgeable of the medical condition in question, must also be consulted. In 2014, an amendment to the law expanded euthanasia to include minors, making Belgium the first country to permit euthanasia for children of any age (however, such a change in legislation only concerns minors with the capacity for discernment, which needs to be assessed on a case-by-case basis by the attending physician and a child psychiatrist or psychologist [10]). Other changes have focused on increasing transparency and accountability, particularly emphasised by recent legislative changes on 27 March 2024. Beyond examining each euthanasia registration document, the Federal Commission for the Control and Evaluation of Euthanasia (FCCEE) publishes regular reports to ensure compliance with the law.

Euthanasia is mostly linked to chronic or terminal physical conditions – with a large share due to cancers in terminal phase – but psychiatric disorders also lead to a substantial proportion of euthanasia [11]. Euthanasia for non-terminal illness is a controversial issue and only a few jurisdictions that have implemented assisted dying allow assisted dying for psychiatric disorders or dementia. This is the case in Belgium, but the issue remains controversial and highly debated [12]. It was estimated that, between 2002 and 2021, euthanasia for unbearable suffering caused by psychiatric disorders concerned 370 patients, 1.4% of the total number of euthanasia cases, most of which occurred after 2010. A research based on medical records has found that most (90%) of these were diagnosed more than one disorder [13]. Although concerning a small proportion of the causes of euthanasia in Belgium, psychiatric disorders are often identified to be a cause of concern but data on such a population is sparse [14]. Recent trends in euthanasia for psychiatric disorders show a decline in cases but one reason for such a recent trend might be due to the criminal prosecution and subsequent acquittal of three physicians, including a psychiatrist, for performing euthanasia on a person with a psychiatric illness [15]. Nevertheless, the majority of the increase in cases in Belgium is particularly pronounced for those aged 80 and over, in a nursing home, those having a disease other than cancer and those not expected to die in the near future [7].

Whilst most research and public reports show increasing trends among population sub-groups based on age, gender or the region of residence, they often fail to account for demographic characteristics that explain euthanasia trends. A public health approach to euthanasia should indeed emphasise the importance considering population-level data and propensities rather than focusing solely on individuals [16]. Ageing population, regional distribution, the higher share of women in the older age groups can significantly impact the incidence of euthanasia cases. For example, older age groups may have higher euthanasia rates due to the prevalence of terminal illnesses, while regional differences might reflect varying access or cultural attitudes towards euthanasia [5, 17]. Failing to account for these characteristics could lead to skewed results.

Such a demographic approach has not been widely used yet but a few studies have already considered euthanasia incidence among population sub-groups. Differences have been observed across gender. Belgian official data show a relatively equal distribution across genders with 49.6% of female in 2020 [8] and data on euthanasia as the ratio on all deaths by gender show similar rates among genders [18]. However, differences were identified when looking at the specific conditions justifying the euthanasia as female are overrepresented in psychiatric cases [19, 20], although representing a small share of all cases. Similarly, studies have shown regional differences. In the Netherlands, unexplained geographical variation in the incidence of euthanasia was observed across provinces [1]. Age, church attendance, political orientation, income, self-experienced health and availability of voluntary workers were associated with such differences, but a large part of the gap remained unexplained. In Belgium, official figures show higher propensities in the Flemish region [21] and most research focuses on the Flanders [22, 23]. The Belgian Federal Commission for the Control and Evaluation of Euthanasia attributes this in part of different socio-cultural attitudes and different medical practices in the North and the South of the country [8]. For instance, it was suggested, in 2009, that physicians in French-speaking Belgium perform palliative sedation more than twice as often as their counterparts in the north of the country [24].

Focusing on administrative data gathered by the Belgian Federal Commission for the Control and Evaluation of Euthanasia (FCCEE), this study has three objectives. First, to provide incidence rates of euthanasia by age, gender, region and health condition over the last decade (2014 − 2013). Second, to compare these figures with population characteristics and changes and to provide weighted incidence rates adjusting for demographic characteristics. Third, to provide information on changes observed over the last ten years for specific population sub-groups.

## Methods

### Data

We use data routinely gathered by the Federal Commission for the Control and Evaluation of Euthanasia (*FCCEE*). Data are derived from the individual reports submitted by euthanasia practitioners. They are fully anonymised and include all reported euthanasia cases since 2004 with information on the reasons for euthanasia, gender, age-group and language. We additionally use open data on population provided by *Statbel*, the Belgian Office for Statistics. These data aggregate different figures from administrative sources to gather population sizes by area of residence, gender and age.

### Euthanasia variables

The study focuses on five variables available within the FCCEE dataset:

*Reasons for euthanasia*: The Federal Commission for the Control and Evaluation of Euthanasia (FCCEE) identifies eleven possible medical conditions for euthanasia: (1) tumours and cancers; (2) polypathology (multimorbidity); (3) nervous system diseases (NSD); (4) circulatory system-related diseases; (5) psychiatric disorders; (6) respiratory diseases; (7) cognitive disorders; (8) bones or muscle diseases; (9) traumatic injuries; (10) digestive diseases; (11) other types of non-classified causes. In this study, we avoid using reasons that concerned < 1% of all euthanasia cases and distinguish seven modalities: (1) cancer and tumours; (2) multimorbidity; (3) nervous system diseases; (4) circulatory system-related diseases; (5) psychiatric disorders; (6) cognitive disorders and (7) others (i.e. respiratory diseases; bones or muscle diseases; traumatic injuries; digestive diseases; other types of non-classified causes). We follow the distinction made by the FCCEE between psychiatric disorders and cognitive disorders because they relate to different conditions and different profiles. We use ‘polypathology’ as the reference category. This is defined by the FCCEE at the coexistence of multiple (> 1) chronic disease. This is referred as “multimorbidity” throughout the study.

*Age group*: FCCEE data contains information on the birth date and the death date. In this study, we use eight age bands post-calculated by the FCCEE to keep the cases anonymous: 15–29, 30–39, 40–49, 50–59 (reference category), 60–69, 70–79, 80–89 and 90 and over.

*Gender*: FCCEE data contains information on gender as reported by the medical practitioner that includes two modalities, male or female (reference category).

*Language*: Belgium is a Federal State with three regions (Wallonia, Flanders and Brussels). Until recently, the place of residence (at province or region level) was not systematically collected by the FCCEE making the interpretation of the place of residence complex over a time span of 10 years. However, data systematically include information on the language used by the medical practitioner to report euthanasia cases. We use this information to impute regional difference by distinguishing euthanasia reported by a Dutch-speaking or French-speaking practitioner (reference category).

*Year*: The dataset includes information about the year of euthanasia, i.e., the year when the act was completed and is coded from 1 (2014) to 10 (2023).

### Population variables

We generate population weights based on demographic data retrieved from the Belgian Statistical Office (*Statbel*). This data includes information on the total population as of January 1 st for each selected year (2014 to 2023), broken down by age group, sex, and region of residence. We chose to use population figures instead of the number of deaths by sub-group, as done in previous studies [1], because a non-negligible share of euthanasia is performed on patients not expected to die in the foreseeable future, including those with dementia or psychiatric disorders – 14.4% of all cases in 2020–2021 [8]. The weights are calculated for each line of euthanasia counts by year, age, gender, and language, and are then applied to the different reasons for euthanasia. The weight is calculated as the ratio between the population of a specific subgroup and the total population aged 15 and over for the selected years. Demographic data do not include information on language. To tackle this issue, the French-speaking population was calculated as the sum of the population residing in Wallonia and 90% of the population in Brussels and the Dutch speaking as the sum of the Flanders residents and 10% of the Brussels population, reflecting the Belgian language repartition. We additionally do *sensitivity analyses* using only Wallonia and Flanders and excluding Brussels. Descriptive statistics on population composition in Belgium from 2014 to 2023 is shown in *supplementary file S1.*

### Analyses

We conducted a Poisson fixed effects analysis on the count data of euthanasia cases in Belgium, examining the effects of year, age group, gender, language, and reason for euthanasia. Since the study examines the total number of euthanasia cases over time – a count variable that has shown an increasing trend – we apply a Poisson model, which is specifically designed for non-negative integer outcomes and accounts for the mean-variance relationship inherent in such data [25], as done previously on suicide count data [26, 27]. We exponentiate the coefficients to obtain the incidence rate ratio (IRR) [28, 29]. Although the dataset comprises all reported euthanasia cases, 95% confidence intervals (95%CI) are presented to convey the precision of the estimates.

We compare two models: an unweighted model and a weighted model that adjusts for demographic characteristics. The *unweighted model* provides a baseline analysis, directly examining the relationship between euthanasia counts and the predictor variables including the year of death, reasons for euthanasia, age group, gender and language/region. This model assumes that the observed counts are representative of the entire population without considering demographic differences. The unweighted model is useful for identifying broad trends and associations in the data but may overlook the influence of demographic patterns, potentially leading to biased results. We then introduce a *weighted model* adjusting the analysis based on the distribution of age groups, gender, and regions for each year – as suggested by Lienfeld & Pyne [30]. By weighting the data, the model ensures that each demographic group’s contribution to the euthanasia counts is proportionate to its representation in the population. For instance, as the population ages, the elderly may account for a larger share of euthanasia cases. The weighted model adjusts for this by giving more weight to these groups, thus offering a more accurate reflection of their influence. Similarly, regional, age groups or gender differences that might be underrepresented in the raw counts are corrected in the weighted model, preventing skewed interpretations. We additionally run a model including both the unweighted and weighted models including an interaction term between each explanatory variable and the type of model to calculate the *difference* between the weighted and unweighted models. Results for the difference analysis correspond to the ratio between the weighted and unweighted models.

Comparing the weighted and unweighted models allows us to explore how demographic adjustments affect the analysis of euthanasia counts. The unweighted model provides insights into the raw associations between variables, while the weighted model reveals how these relationships change when demographic factors are considered. If the coefficient in the unweighted model is higher than in the weighted model, it indicates that the incidence rate is higher than expected. Conversely, if the unweighted coefficient is lower, it suggests an underrepresentation of the specific variable in the incidence rate. We replicate the unweighted and weighted models using a simple fully adjusted model as well as using multiplicative interaction terms between the year of death and, respectively, reasons for euthanasia, age group, gender and language/region.

We include several additional analyses. First, we provide sensitivity checks using the Belgium population restricted to Wallonia and Flanders. Second, to address changes over a 10-year span, we conduct a counterfactual analysis by comparing the weighted model with a model where weights are kept at baseline levels (2014), and we calculate the difference between these models. This approach allows us to disentangle differences due to demographic changes over time from those that are not related to time but demographic composition as such. Third, to address the incidence of euthanasia by gender, we include a two-way interaction between gender and reason for euthanasia.

The software R version 4.2.3 was used for all the analyses. We specifically used the glm command, which is a core R package for generalised linear models (including Poisson regression) and the effects package [31] a well as the core package ggplot2 to display model predictions.

## Results

### Descriptive statistics

Table 1 exhibits the reported cases of euthanasia by year, reason for euthanasia, age group, gender and language and unweighted and weighted percentages. Additional descriptive statistics using the weights excluding the Brussels population are shown in *supplementary file S.2*.

**Table 1:** Reported cases of euthanasia by year, reason for euthanasia, age group, gender and Language and unweighted and weighted percentages

		*N*	Percentage	Weighted percentage			*N*	Percentage	Weighted percentage
Year	2014	1,928	7.76	7.95	Age group	15–29	88	0.35	0.78
	2015	2,021	8.14	8.55		30–39	237	0.95	1.51
	2016	2,028	8.16	8.41		40–49	676	2.72	4.46
	2017	2,313	9.31	9.32		50–59	2,182	8.78	15.50
	2018	2,359	9.50	9.47		60–69	5,027	20.24	30.17
	2019	2,657	10.70	10.77		70–79	6,830	27.50	28.61
	2020	2,445	9.84	10.00		80–89	7,048	28.37	17.39
	2021	2,700	10.87	10.86		90 and over	2,752	11.08	1.57
	2022	2,966	11.94	11.45		Total	24,840	100	100
	2023	3,423	13.78	13.21					
	Total	24,840	100	100					
					Gender	Female	12,486	50.27	51.26
Reason for euthanasia	Multimorbidity	4,257	17.14	11.22		Male	12,354	49.73	48.74
	Dementia	244	0.98	0.97		Sum	24,840	100	100
	NSD	2,352	9.47	9.48					
	Others	303	1.22	1.30					
	Psychiatric disorders	318	1.28	1.99	Language	FR	6,097	24.55	17.10
	Specific diseases	2,219	8.93	7.23		NL	18,743	75.45	82.90
	Cancers	15,147	60.98	67.81		Total	24,840	100	100
	Total	24,840	100	100					

The table shows that, from the 1^st^ of January 2013 to the 3^1st^ of December 2023, the total number of reported euthanasia cases was 24.840. The percentage distribution across years shows an increasing trend with 7.76% of the cases occurring in 2014 and 13.78 in 2023. Rates have constantly increased except at the start of the COVID-19 pandemic, in 2020, where they have slightly dropped. Looking at the percentages weighted for demographic composition, the trend remains similar but with a smaller decrease during the pandemic. Cancers is the main reason for getting euthanasia as it concerns 60.98% of all reported cases (67.81% when data are weighted). Multimorbidity and NSD respectively account for 17.14 and 9.47% of the cases, 11.22 and 9.48 when data are weighted. Psychiatric disorders and dementia account for 1.28 and 0.98 of the total euthanasia, 1.99 and 0.97 when data are weighted. The age group decomposition shows that euthanasia cases are over-represented in the oldest age groups with 28.37 and 11.08% of the cases occurring at ages 80–89 and 90 and over respectively. Weighted for demographic composition, these cases account for respectively 17.39 and 1.57 per cent of the total. By contrast, they are under-represented in the youngest generation with, for instance, 20.27% of the cases reported in the 60–69 age group whilst weighted data show a percentage of 30.17. Unweighted data show that female are slightly more represented within the dataset (50.27%) compared to male. However, weighted data show a percentage of 51.26, meaning that men are slightly more represented in the euthanasia cases after adjusting for demographic statistics. Finally, euthanasia cases are more often observed when reported by Dutch-speaking medical practitioners (3/4th of the cases). Demographic weights balance this observation by reducing the percentage of French-speaking euthanasia by seven per cent but not reversing the trend.

### Weighted and unweighted incidence rates

Table 2 shows the results of the Poisson fixed effects including the main model [model 1] and the interactions between year and reason [2], age group [3], gender [4] and language/region [5]. The estimates from these five models are shown in *supplementary file S.3*.

**Table 2:** Incidence rate ratios (IRR) and 95%CI of euthanasia cases in the weighted and unweighted datasets, main model and interaction terms

		Unweighted	Weighted	Difference			Unweighted	Weighted	Difference
**[Model 1]**		**Main model**							
Year		1.061	1.044	0.984		Specific diseases	0.894	0.904	1.012
		(1.056–1.066)	(1.039–1.049)	(0.978–0.991)			(0.878–0.910	(0.884–0.925)	(0.982–1.042)
Age group	15–29	0.04	0.041	1.015		Cancers	0.909	0.912	1.004
		(0.032–0.050)	(0.035–0.048)	(0.781–1.327)			(0.898–0.920	(0.898–0.927)	(0.984–1.024)
	30–39	0.109	0.109	1.007					
		(0.095–0.124)	(0.097–0.122)	(0.844–1.202)					
	40–49	0.310	0.311	1.002	[Model 3]		Year^*^ Age group		
		(0.284–0.338)	(0.289–0.334)	(0.896–1.122)					
	60–69	2.304	2.300	0.998	Year		1.015	1.009	0.994
		(2.191–2.423)	(2.204-2.400)	(0.934–1.066)			(1.000-1.030)	(0.997–1.021)	(0.975–1.013)
	70–79	3.130	3.132	1.001	Age group	15–29	0.044	0.043	0.988
		(2.983–3.285)	(3.001–3.271)	(0.938–1.067)			(0.027–0.068)	(0.030–0.060)	(0.562–1.767)
	80–89	3.230	3.281	1.016		30–39	0.092	0.091	0.982
		(3.079–3.390)	(3.128–3.441)	(0.949–1.087)			(0.068–0.124)	(0.069–0.117)	(0.659–1.469)
	90+	1.601	2.203	1.376		40–49	0.315	0.318	1.008
		(1.513–1.694)	(1.962–2.465)	(1.210–1.561)			(0.261–0.380)	(0.272–0.371)	(0.789–1.288)
Gender	Male	0.934	1.076	1.152		60–69	1.962	1.919	0.978
		(0.911–0.958)	(1.046–1.105)	(1.110–1.195)			(1.756–2.195)	(1.746–2.110)	(0.845–1.132)
Language	NL	3.196	3.058	0.957		70–79	2.299	2.286	0.994
		(3.105–3.290)	(2.949–3.171)	(0.914–1.002)			(2.065–2.563)	(2.075–2.519)	(0.860–1.149)
Reason	Dementia	0.057	0.086	1.503		80–89	2.475	2.497	1.009
		(0.050–0.065)	(0.074–0.099)	(1.237–1.824)			(2.224–2.756)	(2.243–2.781)	(0.867–1.174)
	NSD	0.524	0.834	1.590		90+	0.875	1.213	1.386
		(0.498–0.551)	(0.785–0.886)	(1.470–1.720)			(0.767–0.999)	(0.896–1.622)	(0.996–1.908)
	Others	0.071	0.116	1.634	Year * Age group	15–29	0.986	0.991	1.005
		(0.063–0.080)	(0.102–0.132)	(1.375–1.940)			(0.916–1.063)	(0.938–1.047)	(0.916–1.102)
	Psychiatric disorders	0.075	0.178	2.378		30–39	1.029	1.034	1.005
		(0.067–0.084)	(0.160–0.197)	(2.038–2.778)			(0.982–1.078)	(0.993–1.076)	(0.945–1.069)
	Specific diseases	0.521	0.644	1.236		40–49	0.997	0.995	0.999
		(0.495–0.549)	(0.604–0.688)	(1.138–1.343)			(0.967–1.027)	(0.971–1.021)	(0.960–1.039)
	Cancers	3.760	6.129	1.630		60–69	1.028	1.033	1.004
		(3.635–3.891)	(5.867–6.407)	(1.542–1.723)			(1.010–1.047)	(1.017–1.048)	(0.981–1.027)
						70–79	1.054	1.056	1.001
							(1.037–1.072)	(1.040–1.072)	(0.979–1.024)
[Model 2]		Year * Reason				80–89	1.047	1.049	1.002
							(1.029–1.065)	(1.031–1.066)	(0.978–1.026)
Year		1.145	1.129	0.986		90+	1.105	1.102	0.997
		(1.133–1.158)	(1.113–1.146)	(0.968–1.004)			(1.084–1.127)	(1.057–1.149)	(0.952–1.044)
Reason	Dementia	0.063	0.077	1.207					
		(0.045–0.087)	(0.052–0.111)	(0.727–1.993)					
	NSD	0.671	1.047	1.561	[Model 4]		Year * Gender		
		(0.591–0.761)	(0.901–1.218)	(1.282-1.900)					
	Others	0.154	0.232	1.505	Year		1.065	1.05	0.986
		(0.119–0.199)	(0.174–0.306)	(1.025–2.207)			(1.058–1.072)	(1.043–1.057)	(0.977–0.995)
	Psychiatric disorders	0.195	0.471	2.415	Gender	Male	0.977	1.142	1.17
		(0.152–0.247)	(0.374–0.589)	(1.734–3.372)			(0.922–1.035)	(1.073–1.217)	(1.074–1.274)
	Specific diseases	1.040	1.196	1.150	Year * Gender	Male	0.993	0.990	0.997
		(0.921–1.174)	(1.024–1.395)	(0.945–1.399)			(0.984–1.001)	(0.980–0.999)	(0.984–1.010)
	Cancers	6.816	10.80	1.584					
		(6.257–7.432)	(9.669–12.084)	(1.377–1.825)					
Year * Reason	Dementia	0.985	1.018	1.034	[Model 5]		Year * Language		
		(0.940–1.032)	(0.966–1.074)	(0.963–1.110)					
	NSD	0.962	0.965	1.003	Year		1.11	1.098	0.988
		(0.945–0.980)	(0.944–0.986)	(0.975–1.031)			(1.101–1.120)	(1.085–1.111)	(0.974–1.003)
	Others	0.881	0.893	1.014	Language	NL	4.613	4.391	0.952
		(0.846–0.917)	(0.854–0.933)	(0.954–1.076)			(4.301–4.951)	(4.022–4.798)	(0.850–1.066)
	Psychiatric disorders	0.851	0.847	0.995	Year * Language	NL	0.942	0.942	1
		(0.818–0.886)	(0.816–0.879)	(0.943–1.051)			(0.932–0.951)	(0.930–0.954)	(0.984–1.017)

**Fig. 1:**
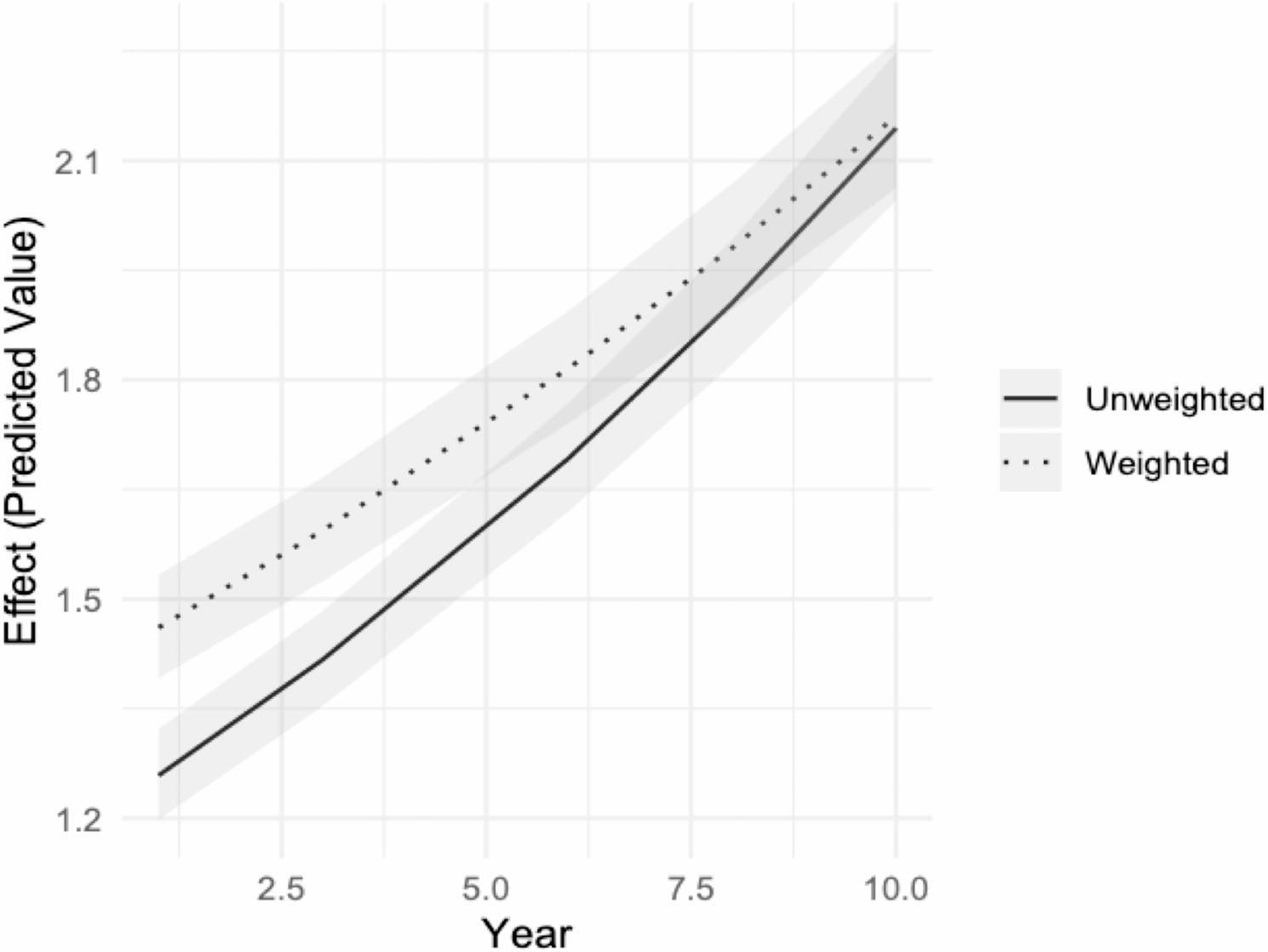
Comparison of estimated differences in years in the weighted and unweighted models

In the main model, we observe that the contribution of time (in years) in explaining euthanasia cases is lower – by 98.4% – in the weighted model [1.044 (95%CI = 1.039;1.049)] compared to the unweighted model [1.061(95%CI = 1.056;1.066)]. In other words, the raw data show an annual increase of 6.1% of the incidence rate against 4.4 in the adjusted model. This suggests that the unweighted model might overestimate the impact of time on the euthanasia rate because it does not account for population’s demographics. When demographic factors are considered, the influence of time is somewhat reduced, indicating that part of the observed increase in euthanasia rates over time in the unweighted model could be due to demographic composition rather than time itself. Figure 1 shows the predicted incidence rates of time in both models, illustrating such a sharper slope in the unweighted model compared to the model adjusting for demographic characteristics and change.

The opposite is observed for the 90 and over age group (compared to the 50–59 reference). The unweighted model shows an incidence of 1.601 (95%CI = 1.513;1.694) but accounting for demographic composition shows that the contribution is much higher (2.203 (95%CI = 1.962;2.465), a ratio of 1.376. The same is observed for the male population (compared to the female population). The incidence rate is 0.934 (95%CI = 0.911;0.958) in the unweighted model but the weighted model shows a coefficient of 1.076 (95%CI = 1.046;1.105) indicating that, after adjusting for demographic composition and change, more male than female received euthanasia. Regional differences remain in the unweighted [3.196 (95%CI = 3.105 3.290)] and weighted model [3.058 (95%CI = 2.949;3.171)] but adjusting for demographic composition slightly reduces the incidence amongst the Dutch-speaking group. Finally, the nature of contribution of the reasons explaining euthanasia does not change but their intensity does. Cancers is the principal reason for euthanasia but its contribution to euthanasia case is doubled (by 1.63) when adjusting for demographic composition. Similarly, psychiatric disorders show a higher contribution when data are weighted, which reflects that these cases concern younger age groups.

**Fig. 2:**
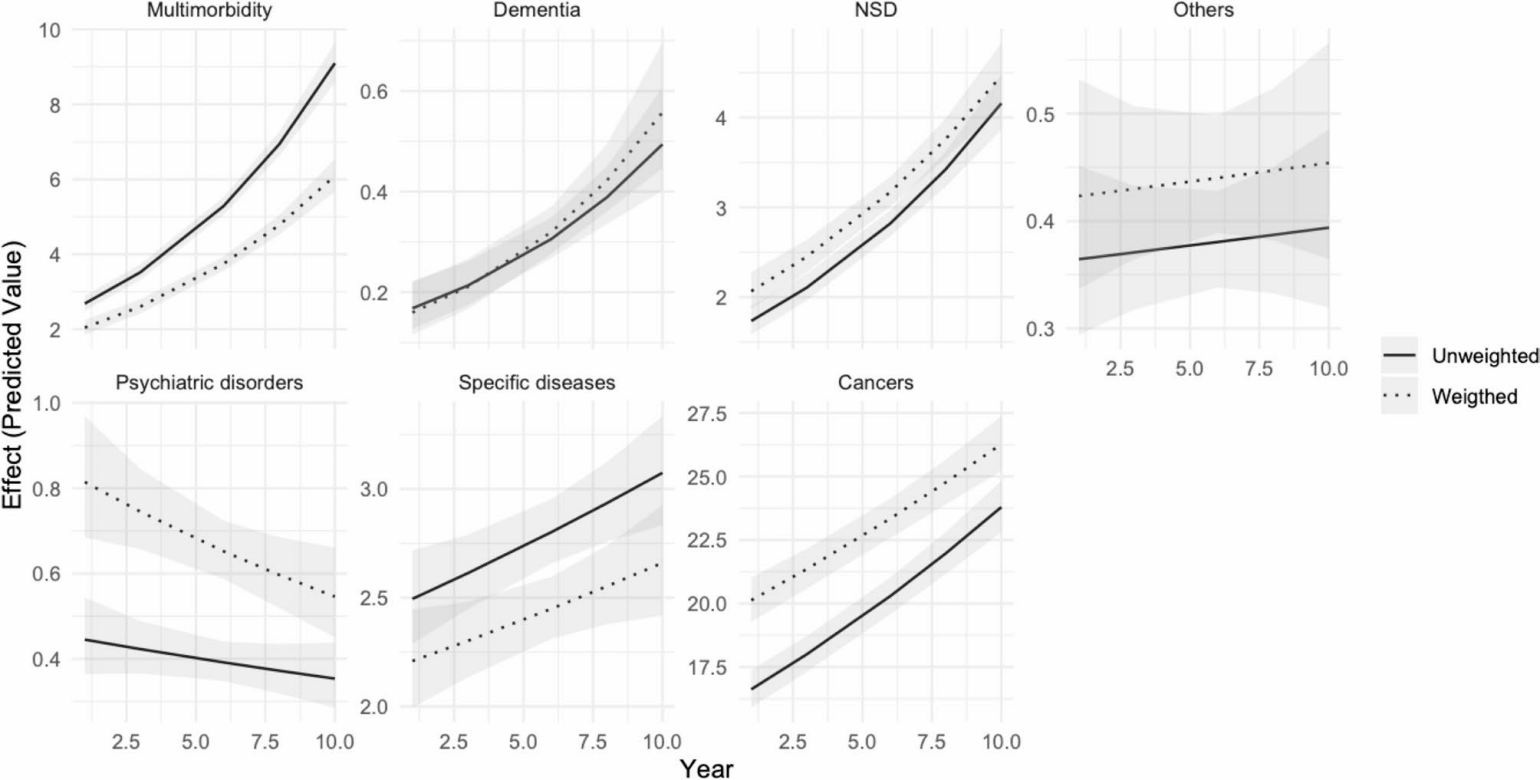
Comparison of estimated differences in years for euthanasia for reason of euthanasia in the weighted and unweighted models

Models [2–5] explore changes over time. Model 2 shows that, compared to multimorbidity, the incidence of euthanasia for other health reasons has generally declined over the period, with the exception of dementia in the weighted model, where the slope for dementia is steeper when adjusting for demographic composition. This trend is more clearly illustrated in Fig. 2, which plots the predicted incidence rates for each reason. Multimorbidity has seen the most significant increase over time. In contrast, the IRR of euthanasia for psychiatric disorders relative to euthanasia for multimorbidity has not risen over the past ten years; in fact, there is a slight declining trend, although this should be interpreted cautiously due to the small number of cases. Model 3 examines the interaction between age groups and years. The interaction term’s difference between the weighted and unweighted models hovers around 1, indicating minimal variation over time when adjusting for demographic composition. Model 4 looks at trends in genders and confirms earlier observations that adjusting for demographic composition increases the incidence of euthanasia amongst male by 17%. However, little change over time is observed, particularly in the weighted model [0.990 (95%CI = 0.980;0.999]. Finally, Model 5 explores changes over time by language/region. The main effect confirms that the incidence of euthanasia is higher among the Dutch-speaking population [4.613 (95%CI = 4.301;4.951)]. Adjusting for demographic composition slightly reduces this incidence [4.391 (95%CI = 4.022;4.798] but does not reverse the overall trend. The interaction effect reveals that, over time, there has been a slight decrease in Dutch-speaking cases [unweighted = 0.942 (95%CI = 0.932;0.951), weighted = 0.942 (95%CI = 0.930;0.954)] relative to French-speaking cases, a decrease that is not explained by demographic composition or changes in it.

The study includes additional analyses. First, we conducted a sensitivity check by restricting the region of residence to the Flemish and Walloon regions and excluding Brussels. This analysis showed no significant differences in the estimates, as detailed in *Supplementary File S.4*.

Second, we generated a counterfactual model by applying the baseline weight from 2014 to all subsequent years and comparing it with the original weight. The results, provided in *Supplementary Files S.5 and S.6* using both full and restricted weights, show minimal differences between the original and counterfactual models. This suggests that the differences observed earlier are due to demographic composition rather than demographic change. Given the short time period considered, this result is expected; a longer time period would likely increase the differences between the two models. Some differences are observed for the health conditions. The weighted model indicates an IRR of 0.834 (95%CI = 0.785;0.886), 0.178 (95%CI = 0.160;0.197) and 6.129 (95%CI = 5.867;6.407) respectively from NSD, psychiatric disorders and cancers. These would be 0.844 (95%CI = 0.794;0.898), 0.187 (95%CI = 0.168;0.208) and 0.649 (95%CI = 0.607;0.694) if population composition were kept constant at baseline levels. In other words, not accounting for demographic change over time would lead to overestimate the incidence of these conditions respectively by 1.2, 5.5 and 1.1%.

**Fig. 3:**
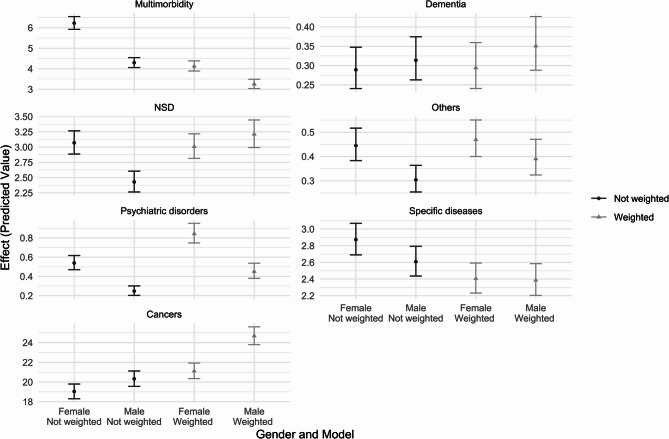
Interaction between the reason for euthanasia and gender in the unweighted and weighted models

Third, we run the model including an interaction term between gender and health conditions, controlling for time. Estimates are shown in *supplementary file S.7.* whilst main effects are plotted in Fig. 3. What can be observed is that adjusting for population demographics using the weighting technique change the gender gap in reasons for euthanasia that is observed. For instance, looking at NSD reasons, we observe a higher incidence within female in the unweighted population. After adjustment for population characteristics, incidence is relatively similar across genders. By contrast, adjusting for demographic composition increases the incidence of psychiatric disorders amongst females and the incidence of cancers amongst male.

## Discussion

More research is needed to thoroughly understand assisted dying prevalence in countries that have implemented it [6]. This is particularly relevant as some countries are currently debating the potential implementation of some assisted dying schemes [32]. With euthanasia figures increasing in all states that have implemented such legislation, a major question is understanding the demographic characteristics underlying the incidence rate. Factors such as an ageing population, the unbalanced gender distribution in older age, and regional differences all play a role in explaining euthanasia figures and trends. Using administrative data on both euthanasia counts and population demographics, this study is one of the first [33] to point out the necessity to balance descriptive statistics. It is not, however, without limitations.

A first limitation is about the type of data available on euthanasia in Belgium. The FCCEE routinely collects and assesses information forms from practitioners but no information on patients’ national insurance number (i.e., national social security number) is collected. Consequently, no merging is possible between, for instance, FCCEE data and social security information such as incomes. This would be a capital information to address potential gaps in socio-economic status among euthanasia patients, but such a perspective is currently impossible. A second limitation is about data collected by the FCCEE. Until 2024, the registration document was partially anonymized, which meant that no data on the patients’ region of residence (province) was available. Only information on euthanasia report language (French or Dutch) was routinely collected. Because residency information was not routinely collected, no reliable information is available on foreign residents who got euthanasia in Belgium; however, we estimate that this figure is below 1% of all euthanasia cases. A third limitation is about the population propensities of specific health condition. One ideal type of control would be to include information on the causes of deaths within sub-populations and to match them with euthanasia cases by reasons. However, this is not possible because the FCCEE uses its own nomenclature and the large share of patients receiving euthanasia because of multimorbidity makes it impossible. Additionally, we restricted our study to the years 2014–2023 because a large part of the increase in euthanasia cases was observed over the period, particularly euthanasia for psychiatric disorders. Finally, this study focuses only on reported cases whilst a substantial number of assisted dying occur without being reported as euthanasia [22, 34].

Nevertheless, five main findings flow from this study.

First, the increasing trend observed in Belgium is slightly balanced by demographic composition. The number of cases has increased by about 4% per years. This is large but not compared to gross figures where it is 6%.

Second, euthanasia for psychiatric disorders is under the spotlight whilst it concerns a small number of people yearly and the rate of change, compared, for instance, to euthanasia for multimorbidity, has not drastically increased.

Third, euthanasia cases justified by cancers have increased but a share of the increase is due to both population composition and population ageing, contributing to an increase in the total number of cases over the past ten years.

Fourth, gender differences are due to both population demographics (more female at older age) and specific health condition propensities. We were able to control for demographics, not for condition propensities. Nevertheless, differences exist with more female receiving euthanasia for psychiatric disorders and more male receiving euthanasia for cancers. These differences should be investigated further.

Finally, accounting for demographic characteristics very slightly reduces the regional divide in Belgium but a large gap remains between Wallonia and the Flanders, illustrating different euthanasia cultures in the North and the South of the country.

## Conclusion

Considering demographic characteristics in euthanasia research is crucial for obtaining accurate and meaningful results. This approach ensures that the analysis reflects the true population structure, leading to more reliable interpretations.

## Supplementary Information

Supplementary Material 1.

## Data Availability

Data is available upon request to the Federal Commission for the Control and Evaluation of Euthanasia (FCCEE).

## Abbreviations

95%CI: 95% Confidence Interval
FCCEE: Belgian Federal Commission for the Control and Evaluation of Euthanasia
IRR: Incidence Rate Ratio
NSD: Nervous system diseases
Statbel: The Belgian Statistical Office
