# Supplementary material for: Incidence of reported cases of euthanasia adjusted for demographic composition: a study of ten years of Belgian administrative data (2014–2023)
Source: BMC Public Health. 2025 Nov 7;25:3843. doi: 10.1186/s12889-025-24839-x (PMC12595677; doi:10.1186/s12889-025-24839-x)
Supplement: Supplementary file 1 — Supplementary Material 1. [file 12889_2025_24839_MOESM1_ESM.docx]

**Supplementary files**

[Supplementary file S1. Demographic changes in Belgium between 2014 and 2024 3](#_Toc187429087)

[Supplementary file S1.1. Age group pyramid by year, gender and region (total figures) 3](#_Toc187429088)

[Supplementary file S1.2. Age group pyramid by year, gender and region (percentage by year) 4](#_Toc187429089)

[Supplementary file S2. Descriptive statistics 5](#_Toc187429090)

[Supplementary file S2.1. Reported cases of euthanasia by year, reason for euthanasia, age group, gender and language and unweighted and weighted percentages. 5](#_Toc187429091)

[Supplementary file S2.1. Reported cases of euthanasia by year, reason for euthanasia, age group, gender and language and unweighted and weighted percentages. Sensitivity check with weight calculated on Flanders and Wallonia only. 6](#_Toc187429092)

[Supplementary file S3. Comparison of the unweighted and weighted Poisson regressions and estimated differences across models – original weight 7](#_Toc187429093)

[Supplementary file S3.1. Main model (no interaction) 7](#_Toc187429094)

[Supplementary file S3.2. Interaction between year and reason for euthanasia 9](#_Toc187429095)

[Supplementary file S3.3. Interaction between year and age group 11](#_Toc187429096)

[Supplementary file S3.4. Interaction between year and gender 13](#_Toc187429097)

[Supplementary file S3.5. Interaction between year and reason/language 15](#_Toc187429098)

[Supplementary file S4. Comparison of the unweighted and weighted Poisson regressions and estimated differences across models – sensitivity checks using weight restricted to Flanders and Wallonia 17](#_Toc187429099)

[Supplementary file S4.1. Main model (no interaction) 17](#_Toc187429100)

[Supplementary file S4.2. Interaction between year and reason for euthanasia 19](#_Toc187429101)

[Supplementary file S4.3. Interaction between year and age group 21](#_Toc187429102)

[Supplementary file S4.4. Interaction between year and gender 23](#_Toc187429103)

[Supplementary file S4.5. Interaction between year and reason/language 25](#_Toc187429104)

[Sensitivity file S5. Counter factual analysis 27](#_Toc187429105)

[Supplementary file S6.1. Main model (no interaction) 27](#_Toc187429106)

[Supplementary file S6.2. Interaction between year and reason for euthanasia 29](#_Toc187429107)

[Supplementary file S6.3. Interaction between year and age group 31](#_Toc187429108)

[Supplementary file S6.4. Interaction between year and gender 34](#_Toc187429109)

[Supplementary file S6.5. Interaction between year and reason/language 36](#_Toc187429110)

[Sensitivity file S6. Counter factual, sensitivity check on Flanders and Wallonia populations only 38](#_Toc187429111)

[Supplementary file S6.1. Main model (no interaction) 38](#_Toc187429112)

[Supplementary file S6.2. Interaction between year and reason for euthanasia 40](#_Toc187429113)

[Supplementary file S6.3. Interaction between year and age group 42](#_Toc187429114)

[Supplementary file S6.4. Interaction between year and gender 44](#_Toc187429115)

[Supplementary file S6.5. Interaction between year and reason/language 46](#_Toc187429116)

[Supplementary file S7. Interaction by the reason for euthanasia and gender 48](#_Toc187429117)

# Supplementary file S1. Demographic changes in Belgium between 2014 and 2024

## Supplementary file S1.1. Age group pyramid by year, gender and region (total figures)

## Supplementary file S1.2. Age group pyramid by year, gender and region (percentage by year)

# Supplementary file S2. Descriptive statistics

## Supplementary file S2.1. Reported cases of euthanasia by year, reason for euthanasia, age group, gender and language and unweighted and weighted percentages.

|  |  | **N** | **Percentage** | **Weighted percentage** |  |  |  | **N** | **Percentage** | **Weighted percentage** |
| --- | --- | --- | --- | --- | --- | --- | --- | --- | --- | --- |
| **Year** | 2014 | 1,928 | 7.76 | 7.95 |  | **Age group** | 50-59 | 2,182 | 8.78 | 15.50 |
|  | 2015 | 2,021 | 8.14 | 8.55 |  |  | 15-29 | 88 | 0.35 | 0.78 |
|  | 2016 | 2,028 | 8.16 | 8.41 |  |  | 30-39 | 237 | 0.95 | 1.51 |
|  | 2017 | 2,313 | 9.31 | 9.32 |  |  | 40-49 | 676 | 2.72 | 4.46 |
|  | 2018 | 2,359 | 9.50 | 9.47 |  |  | 60-69 | 5,027 | 20.24 | 30.17 |
|  | 2019 | 2,657 | 10.70 | 10.77 |  |  | 70-79 | 6,830 | 27.50 | 28.61 |
|  | 2020 | 2,445 | 9.84 | 10.00 |  |  | 80-89 | 7,048 | 28.37 | 17.39 |
|  | 2021 | 2,700 | 10.87 | 10.86 |  |  | 90 and over | 2,752 | 11.08 | 1.57 |
|  | 2022 | 2,966 | 11.94 | 11.45 |  |  | Total | 24,840 | 100 | 100 |
|  | 2023 | 3,423 | 13.78 | 13.21 |  |  |  |  |  |  |
|  | Total | 24,840 | 100 | 100 |  |  |  |  |  |  |
|  |  |  |  |  |  | **Gender** | Female | 12,486 | 50.27 | 51.26 |
| **Reason for euthanasia** | Multimorbidity | 4,257 | 17.14 | 11.22 |  |  | Male | 12,354 | 49.73 | 48.74 |
|  | Dementia | 244 | 0.98 | 0.97 |  |  | Sum | 24,840 | 100 | 100 |
|  | NSD | 2,352 | 9.47 | 9.48 |  |  |  |  |  |  |
|  | Others | 303 | 1.22 | 1.30 |  |  |  |  |  |  |
|  | Psychatric disorders | 318 | 1.28 | 1.99 |  | **Language** | FR | 6,097 | 24.55 | 17.10 |
|  | Specific diseases | 2,219 | 8.93 | 7.23 |  |  | NL | 18,743 | 75.45 | 82.90 |
|  | Cancers | 15,147 | 60.98 | 67.81 |  |  | Total | 24,840 | 100 | 100 |
|  | Total | 24,840 | 100 | 100 |  |  |  |  |  |  |

## Supplementary file S2.1. Reported cases of euthanasia by year, reason for euthanasia, age group, gender and language and unweighted and weighted percentages. Sensitivity check with weight calculated on Flanders and Wallonia only.

|  |  | **N** | **Percentage** | **Weighted percentage** |  |  |  | **N** | **Percentage** | **Weighted percentage** |
| --- | --- | --- | --- | --- | --- | --- | --- | --- | --- | --- |
| **Year** | 2014 | 1,928 | 7.76 | 7.97 |  | **Age group** | 50-59 | 2,182 | 8.78 | 15.50 |
|  | 2015 | 2,021 | 8.14 | 8.59 |  |  | 15-29 | 88 | 0.35 | 0.78 |
|  | 2016 | 2,028 | 8.16 | 8.43 |  |  | 30-39 | 237 | 0.95 | 1.51 |
|  | 2017 | 2,313 | 9.31 | 9.35 |  |  | 40-49 | 676 | 2.72 | 4.46 |
|  | 2018 | 2,359 | 9.50 | 9.48 |  |  | 60-69 | 5,027 | 20.24 | 30.17 |
|  | 2019 | 2,657 | 10.70 | 10.81 |  |  | 70-79 | 6,830 | 27.50 | 28.61 |
|  | 2020 | 2,445 | 9.84 | 9.99 |  |  | 80-89 | 7,048 | 28.37 | 17.39 |
|  | 2021 | 2,700 | 10.87 | 10.85 |  |  | 90 and over | 2,752 | 11.08 | 1.57 |
|  | 2022 | 2,966 | 11.94 | 11.38 |  |  | Total | 24,840 | 100 | 100 |
|  | 2023 | 3,423 | 13.78 | 13.15 |  |  |  |  |  |  |
|  | Total | 24,840 | 100 | 10 |  |  |  |  |  |  |
|  |  |  |  |  |  | **Gender** | Female | 12,486 | 50.27 | 51.05 |
| **Reason for euthanasia** | Multimorbidity | 4,257 | 17.14 | 11.21 |  |  | Male | 12,354 | 49.73 | 48.95 |
|  | Dementia | 244 | 0.98 | 0.97 |  |  | Sum | 24,840 | 100 | 100 |
|  | NSD | 2,352 | 9.47 | 9.34 |  |  |  |  |  |  |
|  | Others | 303 | 1.22 | 1.29 |  |  |  |  |  |  |
|  | Psychatric disorders | 318 | 1.28 | 2.02 |  | **Language** | FR | 6,097 | 24.55 | 14.56 |
|  | Specific diseases | 2,219 | 8.93 | 7.21 |  |  | NL | 18,743 | 75.45 | 85.44 |
|  | Cancers | 15,147 | 60.98 | 67.96 |  |  | Total | 24,840 | 100 | 100 |
|  | Total | 24,840 | 100 | 100 |  |  |  |  |  |  |

# Supplementary file S3. Comparison of the unweighted and weighted Poisson regressions and estimated differences across models – original weight

## Supplementary file S3.1. Main model (no interaction)

|  |  | Unweighted | | |  | Weighted | | |  | Difference | | |
| --- | --- | --- | --- | --- | --- | --- | --- | --- | --- | --- | --- | --- |
|  |  | IRR | 95%CI | |  | IRR | 95%CI | |  | IRR | 95%CI | |
|  |  |  | 2.5 % | 97.5 % |  |  | 2.5 % | 97.5 % |  |  | 2.5 % | 97.5 % |
| (Intercept) | | 3.185 | 2.993 | 3.387 |  | 2.252 | 2.105 | 2.408 |  |  |  |  |
| Year | | 1.061 | 1.056 | 1.066 |  | 1.044 | 1.039 | 1.049 |  | 0.984 | 0.978 | 0.991 |
| Age group | 15-29 | 0.040 | 0.032 | 0.050 |  | 0.041 | 0.035 | 0.048 |  | 1.015 | 0.781 | 1.327 |
|  | 30-39 | 0.109 | 0.095 | 0.124 |  | 0.109 | 0.097 | 0.122 |  | 1.007 | 0.844 | 1.202 |
|  | 40-49 | 0.310 | 0.284 | 0.338 |  | 0.311 | 0.289 | 0.334 |  | 1.002 | 0.896 | 1.122 |
|  | 60-69 | 2.304 | 2.191 | 2.423 |  | 2.300 | 2.204 | 2.400 |  | 0.998 | 0.934 | 1.066 |
|  | 70-79 | 3.130 | 2.983 | 3.285 |  | 3.132 | 3.001 | 3.271 |  | 1.001 | 0.938 | 1.067 |
|  | 80-89 | 3.230 | 3.079 | 3.390 |  | 3.281 | 3.128 | 3.441 |  | 1.016 | 0.949 | 1.087 |
|  | 90 and over | 1.601 | 1.513 | 1.694 |  | 2.203 | 1.962 | 2.465 |  | 1.376 | 1.210 | 1.561 |
| Gender | Male | 0.934 | 0.911 | 0.958 |  | 1.076 | 1.046 | 1.105 |  | 1.152 | 1.110 | 1.195 |
| Language | NL | 3.196 | 3.105 | 3.290 |  | 3.058 | 2.949 | 3.171 |  | 0.957 | 0.914 | 1.002 |
| Reason | Dementia | 0.057 | 0.050 | 0.065 |  | 0.086 | 0.074 | 0.099 |  | 1.503 | 1.237 | 1.824 |
|  | NSD | 0.524 | 0.498 | 0.551 |  | 0.834 | 0.785 | 0.886 |  | 1.590 | 1.470 | 1.720 |
|  | Others | 0.071 | 0.063 | 0.080 |  | 0.116 | 0.102 | 0.132 |  | 1.634 | 1.375 | 1.940 |
|  | Psychiatric disorders | 0.075 | 0.067 | 0.084 |  | 0.178 | 0.160 | 0.197 |  | 2.378 | 2.038 | 2.778 |
|  | Specific diseases | 0.521 | 0.495 | 0.549 |  | 0.644 | 0.604 | 0.688 |  | 1.236 | 1.138 | 1.343 |
|  | Cancers | 3.760 | 3.635 | 3.891 |  | 6.129 | 5.867 | 6.407 |  | 1.630 | 1.542 | 1.723 |
|  |  | Null deviance: 74605.7 on 2239 degrees of freedom | | |  | Null deviance: 70353.7 on 2239 degrees of freedom | | |  | Null deviance: 145353 on 4479 degrees of freedom | | |
|  |  | Residual deviance: 7670.7 on 2223 degrees of freedom | | |  | Residual deviance: 4931.5 on 2223 degrees of freedom | | |  | Residual deviance: 12602 on 4446 degrees of freedom | | |
|  |  | AIC: 12854 | |  |  | AIC: 9490.1 | |  |  | AIC: 22345 | |  |

Effects

## Supplementary file S3.2. Interaction between year and reason for euthanasia

|  |  | Unweighted | | |  | Weighted | | |  | Difference | | |
| --- | --- | --- | --- | --- | --- | --- | --- | --- | --- | --- | --- | --- |
|  |  | IRR | 95%CI | |  | IRR | 95%CI | |  | IRR | 95%CI | |
|  |  |  | 2.5 % | 97.5 % |  |  | 2.5 % | 97.5 % |  |  | 2.5 % | 97.5 % |
| (Intercept) | | 1.970 | 1.797 | 2.157 |  | 1.386 | 1.235 | 1.553 |  |  |  |  |
| Year | | 1.145 | 1.133 | 1.158 |  | 1.129 | 1.113 | 1.146 |  | 0.986 | 0.968 | 1.004 |
| Reason | Dementia | 0.063 | 0.045 | 0.087 |  | 0.077 | 0.052 | 0.111 |  | 1.207 | 0.727 | 1.993 |
|  | NSD | 0.671 | 0.591 | 0.761 |  | 1.047 | 0.901 | 1.218 |  | 1.561 | 1.282 | 1.900 |
|  | Others | 0.154 | 0.119 | 0.199 |  | 0.232 | 0.174 | 0.306 |  | 1.505 | 1.025 | 2.207 |
|  | Psychiatric disorders | 0.195 | 0.152 | 0.247 |  | 0.471 | 0.374 | 0.589 |  | 2.415 | 1.734 | 3.372 |
|  | Specific diseases | 1.040 | 0.921 | 1.174 |  | 1.196 | 1.024 | 1.395 |  | 1.150 | 0.945 | 1.399 |
|  | Cancers | 6.816 | 6.257 | 7.432 |  | 10.800 | 9.669 | 12.084 |  | 1.584 | 1.377 | 1.825 |
| Year * Reason | Dementia | 0.985 | 0.940 | 1.032 |  | 1.018 | 0.966 | 1.074 |  | 1.034 | 0.963 | 1.110 |
|  | NSD | 0.962 | 0.945 | 0.980 |  | 0.965 | 0.944 | 0.986 |  | 1.003 | 0.975 | 1.031 |
|  | Others | 0.881 | 0.846 | 0.917 |  | 0.893 | 0.854 | 0.933 |  | 1.014 | 0.954 | 1.076 |
|  | Psychiatric disorders | 0.851 | 0.818 | 0.886 |  | 0.847 | 0.816 | 0.879 |  | 0.995 | 0.943 | 1.051 |
|  | Specific diseases | 0.894 | 0.878 | 0.910 |  | 0.904 | 0.884 | 0.925 |  | 1.012 | 0.982 | 1.042 |
|  | Cancers | 0.909 | 0.898 | 0.920 |  | 0.912 | 0.898 | 0.927 |  | 1.004 | 0.984 | 1.024 |
| Age group | 15-29 | 0.040 | 0.032 | 0.050 |  | 0.041 | 0.035 | 0.048 |  | 0.040 | 0.032 | 0.050 |
|  | 30-39 | 0.109 | 0.095 | 0.124 |  | 0.109 | 0.097 | 0.122 |  | 0.109 | 0.095 | 0.124 |
|  | 40-49 | 0.310 | 0.284 | 0.338 |  | 0.310 | 0.289 | 0.334 |  | 0.310 | 0.284 | 0.338 |
|  | 60-69 | 2.304 | 2.191 | 2.423 |  | 2.300 | 2.204 | 2.400 |  | 2.304 | 2.191 | 2.423 |
|  | 70-79 | 3.130 | 2.983 | 3.285 |  | 3.132 | 3.001 | 3.270 |  | 3.130 | 2.983 | 3.285 |
|  | 80-89 | 3.230 | 3.079 | 3.390 |  | 3.281 | 3.128 | 3.442 |  | 3.230 | 3.079 | 3.390 |
|  | 90 and over | 1.601 | 1.513 | 1.694 |  | 2.197 | 1.956 | 2.458 |  | 1.601 | 1.513 | 1.694 |
| Gender | Male | 0.934 | 0.911 | 0.958 |  | 1.076 | 1.047 | 1.106 |  | 1.152 | 1.110 | 1.195 |
| Language | NL | 3.196 | 3.105 | 3.290 |  | 3.058 | 2.949 | 3.171 |  | 0.957 | 0.913 | 1.002 |
|  |  | Null deviance: 74605.7 on 2239 degrees of freedom | | |  | Null deviance: 70353.7 on 2239 degrees of freedom | | |  | Null deviance: 145353 on 4479 degrees of freedom | | |
|  |  | Residual deviance: 7348.7 on 2217 degrees of freedom | | |  | Residual deviance: 4723.1 on 2217 degrees of freedom | | |  | Residual deviance: 12072 on 4434 degrees of freedom | | |
|  |  | AIC: 12544 | | |  | AIC: 9293.6 | | |  | AIC: 21838 | | |

Effects

## Supplementary file S3.3. Interaction between year and age group

|  |  | Unweighted | | |  | Weighted | | |  | Difference | | |
| --- | --- | --- | --- | --- | --- | --- | --- | --- | --- | --- | --- | --- |
|  |  | IRR | 95%CI | |  | IRR | 95%CI | |  | IRR | 95%CI | |
|  |  |  | 2.5 % | 97.5 % |  |  | 2.5 % | 97.5 % |  |  | 2.5 % | 97.5 % |
| (Intercept) | | 4.117 | 3.724 | 4.544 |  | 2.746 | 2.507 | 3.006 |  |  |  |  |
| Year | | 1.015 | 1.000 | 1.030 |  | 1.009 | 0.997 | 1.021 |  | 0.994 | 0.975 | 1.013 |
| Age group | 15-29 | 0.044 | 0.027 | 0.068 |  | 0.043 | 0.030 | 0.060 |  | 0.988 | 0.562 | 1.767 |
|  | 30-39 | 0.092 | 0.068 | 0.124 |  | 0.091 | 0.069 | 0.117 |  | 0.982 | 0.659 | 1.469 |
|  | 40-49 | 0.315 | 0.261 | 0.380 |  | 0.318 | 0.272 | 0.371 |  | 1.008 | 0.789 | 1.288 |
|  | 60-69 | 1.962 | 1.756 | 2.195 |  | 1.919 | 1.746 | 2.110 |  | 0.978 | 0.845 | 1.132 |
|  | 70-79 | 2.299 | 2.065 | 2.563 |  | 2.286 | 2.075 | 2.519 |  | 0.994 | 0.860 | 1.149 |
|  | 80-89 | 2.475 | 2.224 | 2.756 |  | 2.497 | 2.243 | 2.781 |  | 1.009 | 0.867 | 1.174 |
|  | 90 and over | 0.875 | 0.767 | 0.999 |  | 1.213 | 0.896 | 1.622 |  | 1.386 | 0.996 | 1.908 |
| Year * Age group | 15-29 | 0.986 | 0.916 | 1.063 |  | 0.991 | 0.938 | 1.047 |  | 1.005 | 0.916 | 1.102 |
|  | 30-39 | 1.029 | 0.982 | 1.078 |  | 1.034 | 0.993 | 1.076 |  | 1.005 | 0.945 | 1.069 |
|  | 40-49 | 0.997 | 0.967 | 1.027 |  | 0.995 | 0.971 | 1.021 |  | 0.999 | 0.960 | 1.039 |
|  | 60-69 | 1.028 | 1.010 | 1.047 |  | 1.033 | 1.017 | 1.048 |  | 1.004 | 0.981 | 1.027 |
|  | 70-79 | 1.054 | 1.037 | 1.072 |  | 1.056 | 1.040 | 1.072 |  | 1.001 | 0.979 | 1.024 |
|  | 80-89 | 1.047 | 1.029 | 1.065 |  | 1.049 | 1.031 | 1.066 |  | 1.002 | 0.978 | 1.026 |
|  | 90 and over | 1.105 | 1.084 | 1.127 |  | 1.102 | 1.057 | 1.149 |  | 0.997 | 0.952 | 1.044 |
| Reason | Dementia | 0.057 | 0.050 | 0.065 |  | 0.086 | 0.074 | 0.099 |  | 1.503 | 1.237 | 1.824 |
|  | NSD | 0.524 | 0.498 | 0.551 |  | 0.834 | 0.785 | 0.886 |  | 1.590 | 1.470 | 1.720 |
|  | Others | 0.071 | 0.063 | 0.080 |  | 0.116 | 0.102 | 0.132 |  | 1.634 | 1.375 | 1.940 |
|  | Psychiatric disorders | 0.075 | 0.067 | 0.084 |  | 0.178 | 0.160 | 0.197 |  | 2.378 | 2.038 | 2.778 |
|  | Specific diseases | 0.521 | 0.495 | 0.549 |  | 0.644 | 0.604 | 0.688 |  | 1.236 | 1.138 | 1.343 |
|  | Cancers | 3.760 | 3.635 | 3.891 |  | 6.129 | 5.867 | 6.407 |  | 1.630 | 1.542 | 1.723 |
| Gender | Male | 0.934 | 0.911 | 0.958 |  | 1.075 | 1.046 | 1.105 |  | 1.151 | 1.109 | 1.194 |
| Language | NL | 3.196 | 3.105 | 3.290 |  | 3.057 | 2.949 | 3.171 |  | 0.957 | 0.913 | 1.002 |
|  |  | Null deviance: 74605.7 on 2239 degrees of freedom | | |  | Null deviance: 70353.7 on 2239 degrees of freedom | | |  | Null deviance: 145353 on 4479 degrees of freedom | | |
|  |  | Residual deviance: 7537.6 on 2216 degrees of freedom | | |  | Residual deviance: 4852.7 on 2216 degrees of freedom | | |  | Residual deviance: 12390 on 4432 degrees of freedom | | |
|  |  | AIC: 12735 | | |  | AIC: 9425.2 | | |  | AIC: 22161 | | |

Effects

## Supplementary file S3.4. Interaction between year and gender

|  |  | Unweighted | | |  | Weighted | | |  | Difference | | |
| --- | --- | --- | --- | --- | --- | --- | --- | --- | --- | --- | --- | --- |
|  |  | IRR | 95%CI | |  | IRR | 95%CI | |  | IRR | 95%CI | |
|  |  |  | 2.5 % | 97.5 % |  |  | 2.5 % | 97.5 % |  |  | 2.5 % | 97.5 % |
| (Intercept) | | 3.115 | 2.912 | 3.331 |  | 2.186 | 2.032 | 2.351 |  |  |  |  |
| Year | | 1.065 | 1.058 | 1.072 |  | 1.050 | 1.043 | 1.057 |  | 0.986 | 0.977 | 0.995 |
| Gender | Male | 0.977 | 0.922 | 1.035 |  | 1.142 | 1.073 | 1.217 |  | 1.170 | 1.074 | 1.274 |
| Year * Gender | Male | 0.993 | 0.984 | 1.001 |  | 0.990 | 0.980 | 0.999 |  | 0.997 | 0.984 | 1.010 |
| Age group | 15-29 | 0.040 | 0.032 | 0.050 |  | 0.041 | 0.035 | 0.048 |  | 1.015 | 0.781 | 1.327 |
|  | 30-39 | 0.109 | 0.095 | 0.124 |  | 0.109 | 0.097 | 0.122 |  | 1.007 | 0.844 | 1.202 |
|  | 40-49 | 0.310 | 0.284 | 0.338 |  | 0.310 | 0.289 | 0.334 |  | 1.002 | 0.895 | 1.122 |
|  | 60-69 | 2.304 | 2.191 | 2.423 |  | 2.300 | 2.204 | 2.400 |  | 0.998 | 0.935 | 1.066 |
|  | 70-79 | 3.130 | 2.983 | 3.285 |  | 3.133 | 3.001 | 3.271 |  | 1.001 | 0.938 | 1.068 |
|  | 80-89 | 3.230 | 3.079 | 3.390 |  | 3.282 | 3.129 | 3.443 |  | 1.016 | 0.949 | 1.087 |
|  | 90 and over | 1.601 | 1.513 | 1.694 |  | 2.204 | 1.962 | 2.466 |  | 1.377 | 1.210 | 1.561 |
| Reason | Dementia | 0.057 | 0.050 | 0.065 |  | 0.086 | 0.074 | 0.099 |  | 1.503 | 1.237 | 1.824 |
|  | NSD | 0.524 | 0.498 | 0.551 |  | 0.834 | 0.785 | 0.886 |  | 1.590 | 1.470 | 1.720 |
|  | Others | 0.071 | 0.063 | 0.080 |  | 0.116 | 0.102 | 0.132 |  | 1.634 | 1.375 | 1.940 |
|  | Psychiatric disorders | 0.075 | 0.067 | 0.084 |  | 0.178 | 0.160 | 0.197 |  | 2.378 | 2.038 | 2.778 |
|  | Specific diseases | 0.521 | 0.495 | 0.549 |  | 0.644 | 0.604 | 0.688 |  | 1.236 | 1.138 | 1.343 |
|  | Cancers | 3.760 | 3.635 | 3.891 |  | 6.129 | 5.867 | 6.407 |  | 1.630 | 1.542 | 1.724 |
| Language | NL | 3.196 | 3.105 | 3.290 |  | 3.058 | 2.949 | 3.171 |  | 0.957 | 0.914 | 1.002 |
|  |  | Null deviance: 74605.7 on 2239 degrees of freedom | | |  | Null deviance: 70353.7 on 2239 degrees of freedom | | |  | Null deviance: 145353 on 4479 degrees of freedom | | |
|  |  | Residual deviance: 7667.9 on 2222 degrees of freedom | | |  | Residual deviance: 4927.2 on 2222 degrees of freedom | | |  | Residual deviance: 12595 on 4444 degrees of freedom | | |
|  |  | AIC: 12854 | | |  | AIC: 9487.7 | | |  | AIC: 22341 | | |

Effects

## Supplementary file S3.5. Interaction between year and reason/language

|  |  | Unweighted | | |  | Weighted | | |  | Difference | | |
| --- | --- | --- | --- | --- | --- | --- | --- | --- | --- | --- | --- | --- |
|  |  | IRR | 95%CI | |  | IRR | 95%CI | |  | IRR | 95%CI | |
|  |  |  | 2.5 % | 97.5 % |  |  | 2.5 % | 97.5 % |  |  | 2.5 % | 97.5 % |
| (Intercept) | | 2.404 | 2.219 | 2.604 |  | 1.663 | 1.510 | 1.830 |  |  |  |  |
| Year | | 1.110 | 1.101 | 1.120 |  | 1.098 | 1.085 | 1.111 |  | 0.988 | 0.974 | 1.003 |
| Language | NL | 4.613 | 4.301 | 4.951 |  | 4.391 | 4.022 | 4.798 |  | 0.952 | 0.850 | 1.066 |
| Year * Language | NL | 0.942 | 0.932 | 0.951 |  | 0.942 | 0.930 | 0.954 |  | 1.000 | 0.984 | 1.017 |
| Age group | 15-29 | 0.040 | 0.032 | 0.050 |  | 0.041 | 0.035 | 0.048 |  | 1.015 | 0.782 | 1.328 |
|  | 30-39 | 0.109 | 0.095 | 0.124 |  | 0.109 | 0.097 | 0.122 |  | 1.007 | 0.844 | 1.202 |
|  | 40-49 | 0.310 | 0.284 | 0.338 |  | 0.311 | 0.289 | 0.334 |  | 1.002 | 0.896 | 1.122 |
|  | 60-69 | 2.304 | 2.191 | 2.423 |  | 2.301 | 2.205 | 2.401 |  | 0.999 | 0.935 | 1.067 |
|  | 70-79 | 3.130 | 2.983 | 3.285 |  | 3.131 | 2.999 | 3.269 |  | 1.000 | 0.938 | 1.067 |
|  | 80-89 | 3.230 | 3.079 | 3.390 |  | 3.285 | 3.132 | 3.445 |  | 1.017 | 0.950 | 1.088 |
|  | 90 and over | 1.601 | 1.513 | 1.694 |  | 2.206 | 1.964 | 2.468 |  | 1.378 | 1.211 | 1.563 |
| Reason | Dementia | 0.057 | 0.050 | 0.065 |  | 0.086 | 0.074 | 0.099 |  | 1.503 | 1.237 | 1.824 |
|  | NSD | 0.524 | 0.498 | 0.551 |  | 0.834 | 0.785 | 0.886 |  | 1.590 | 1.470 | 1.720 |
|  | Others | 0.071 | 0.063 | 0.080 |  | 0.116 | 0.102 | 0.132 |  | 1.634 | 1.375 | 1.940 |
|  | Psychiatric disorders | 0.075 | 0.067 | 0.084 |  | 0.178 | 0.160 | 0.197 |  | 2.378 | 2.038 | 2.778 |
|  | Specific diseases | 0.521 | 0.495 | 0.549 |  | 0.644 | 0.604 | 0.688 |  | 1.236 | 1.138 | 1.343 |
|  | Cancers | 3.760 | 3.635 | 3.891 |  | 6.129 | 5.866 | 6.406 |  | 1.630 | 1.542 | 1.723 |
| Gender | Male | 0.934 | 0.911 | 0.958 |  | 1.076 | 1.046 | 1.105 |  | 1.152 | 1.110 | 1.195 |
|  |  | Null deviance: 74605.7 on 2239 degrees of freedom | | |  | Null deviance: 70353.7 on 2239 degrees of freedom | | |  | Null deviance: 145353 on 4479 degrees of freedom | | |
|  |  | Residual deviance: 7538.2 on 2222 degrees of freedom | | |  | Residual deviance: 4848.5 on 2222 degrees of freedom | | |  | Residual deviance: 12387 on 4444 degrees of freedom | | |
|  |  | AIC: 12724 | | |  | AIC: 9409 | | |  | AIC: 22133 | | |

Effects

# Supplementary file S4. Comparison of the unweighted and weighted Poisson regressions and estimated differences across models – sensitivity checks using weight restricted to Flanders and Wallonia

## Supplementary file S4.1. Main model (no interaction)

|  |  | Unweighted | | |  | Weighted | | |  | Difference | | |
| --- | --- | --- | --- | --- | --- | --- | --- | --- | --- | --- | --- | --- |
|  |  | IRR | 95%CI | |  | IRR | 95%CI | |  | IRR | 95%CI | |
|  |  |  | 2.5 % | 97.5 % |  |  | 2.5 % | 97.5 % |  |  | 2.5 % | 97.5 % |
| (Intercept) | | 3.185 | 2.993 | 3.387 |  | 2.264 | 2.118 | 2.419 |  |  |  |  |
| Year | | 1.061 | 1.056 | 1.066 |  | 1.043 | 1.038 | 1.048 |  | 0.983 | 0.977 | 0.989 |
| Age group | 15-29 | 0.040 | 0.032 | 0.050 |  | 0.042 | 0.035 | 0.048 |  | 1.029 | 0.794 | 1.343 |
|  | 30-39 | 0.109 | 0.095 | 0.124 |  | 0.110 | 0.098 | 0.123 |  | 1.013 | 0.850 | 1.207 |
|  | 40-49 | 0.310 | 0.284 | 0.338 |  | 0.311 | 0.289 | 0.333 |  | 1.002 | 0.896 | 1.121 |
|  | 60-69 | 2.304 | 2.191 | 2.423 |  | 2.300 | 2.207 | 2.397 |  | 0.998 | 0.936 | 1.065 |
|  | 70-79 | 3.130 | 2.983 | 3.285 |  | 3.136 | 3.008 | 3.269 |  | 1.002 | 0.940 | 1.068 |
|  | 80-89 | 3.230 | 3.079 | 3.390 |  | 3.285 | 3.136 | 3.440 |  | 1.017 | 0.951 | 1.087 |
|  | 90 and over | 1.601 | 1.513 | 1.694 |  | 2.247 | 2.008 | 2.507 |  | 1.404 | 1.238 | 1.588 |
| Gender | Male | 0.934 | 0.911 | 0.958 |  | 1.080 | 1.052 | 1.109 |  | 1.156 | 1.115 | 1.199 |
| Language | NL | 3.196 | 3.105 | 3.290 |  | 3.055 | 2.943 | 3.172 |  | 0.956 | 0.912 | 1.002 |
| Reason | Dementia | 0.057 | 0.050 | 0.065 |  | 0.087 | 0.075 | 0.100 |  | 1.517 | 1.254 | 1.834 |
|  | NSD | 0.524 | 0.498 | 0.551 |  | 0.822 | 0.775 | 0.871 |  | 1.568 | 1.451 | 1.694 |
|  | Others | 0.071 | 0.063 | 0.080 |  | 0.115 | 0.101 | 0.130 |  | 1.614 | 1.362 | 1.912 |
|  | Psychiatric disorders | 0.075 | 0.067 | 0.084 |  | 0.181 | 0.163 | 0.200 |  | 2.418 | 2.077 | 2.817 |
|  | Specific diseases | 0.521 | 0.495 | 0.549 |  | 0.643 | 0.604 | 0.685 |  | 1.234 | 1.138 | 1.339 |
|  | Cancers | 3.760 | 3.635 | 3.891 |  | 6.152 | 5.897 | 6.422 |  | 1.636 | 1.549 | 1.728 |
|  |  | Null deviance: 74605.7 on 2239 degrees of freedom | | |  | Null deviance: 73668.5 on 2239 degrees of freedom | | |  | Null deviance: 148448 on 4479 degrees of freedom | | |
|  |  | Residual deviance: 7670.7 on 2223 degrees of freedom | | |  | Residual deviance: 5126.8 on 2223 degrees of freedom | | |  | Residual deviance: 12797 on 4446 degrees of freedom | | |
|  |  | AIC: 12854 | | |  | AIC: 9860.6 | | |  | AIC: 22715 | | |

Effects

## Supplementary file S4.2. Interaction between year and reason for euthanasia

|  |  | Unweighted | | |  | Weighted | | |  | Difference | | |
| --- | --- | --- | --- | --- | --- | --- | --- | --- | --- | --- | --- | --- |
|  |  | IRR | 95%CI | |  | IRR | 95%CI | |  | IRR | 95%CI | |
|  |  |  | 2.5 % | 97.5 % |  |  | 2.5 % | 97.5 % |  |  | 2.5 % | 97.5 % |
| (Intercept) | | 1.970 | 1.797 | 2.157 |  | 1.399 | 1.250 | 1.562 |  | 1.970 | 1.797 | 2.157 |
| Year | | 1.145 | 1.133 | 1.158 |  | 1.127 | 1.111 | 1.143 |  | 0.985 | 0.940 | 1.032 |
| Reason | Dementia | 0.063 | 0.045 | 0.087 |  | 0.078 | 0.053 | 0.111 |  | 1.226 | 0.747 | 2.003 |
|  | NSD | 0.671 | 0.591 | 0.761 |  | 1.039 | 0.897 | 1.202 |  | 1.548 | 1.276 | 1.878 |
|  | Others | 0.154 | 0.119 | 0.199 |  | 0.230 | 0.173 | 0.301 |  | 1.489 | 1.021 | 2.171 |
|  | Psychiatric disorders | 0.195 | 0.152 | 0.247 |  | 0.481 | 0.386 | 0.597 |  | 2.471 | 1.785 | 3.429 |
|  | Specific diseases | 1.040 | 0.921 | 1.174 |  | 1.189 | 1.024 | 1.381 |  | 1.143 | 0.943 | 1.386 |
|  | Cancers | 6.816 | 6.257 | 7.432 |  | 10.768 | 9.676 | 12.002 |  | 1.580 | 1.377 | 1.814 |
| Year * Reason | Dementia | 0.985 | 0.940 | 1.032 |  | 1.017 | 0.967 | 1.071 |  | 1.033 | 0.964 | 1.107 |
|  | NSD | 0.962 | 0.945 | 0.980 |  | 0.964 | 0.944 | 0.984 |  | 1.002 | 0.974 | 1.030 |
|  | Others | 0.881 | 0.846 | 0.917 |  | 0.892 | 0.855 | 0.932 |  | 1.013 | 0.955 | 1.075 |
|  | Psychiatric disorders | 0.851 | 0.818 | 0.886 |  | 0.846 | 0.816 | 0.876 |  | 0.994 | 0.942 | 1.048 |
|  | Specific diseases | 0.894 | 0.878 | 0.910 |  | 0.905 | 0.885 | 0.925 |  | 1.012 | 0.983 | 1.042 |
|  | Cancers | 0.909 | 0.898 | 0.920 |  | 0.913 | 0.899 | 0.927 |  | 1.005 | 0.985 | 1.025 |
| Age group | 15-29 | 0.040 | 0.032 | 0.050 |  | 0.042 | 0.035 | 0.048 |  | 1.029 | 0.794 | 1.343 |
|  | 30-39 | 0.109 | 0.095 | 0.124 |  | 0.110 | 0.098 | 0.123 |  | 1.013 | 0.850 | 1.207 |
|  | 40-49 | 0.310 | 0.284 | 0.338 |  | 0.310 | 0.289 | 0.333 |  | 1.002 | 0.896 | 1.121 |
|  | 60-69 | 2.304 | 2.191 | 2.423 |  | 2.300 | 2.207 | 2.397 |  | 0.998 | 0.935 | 1.065 |
|  | 70-79 | 3.130 | 2.983 | 3.285 |  | 3.135 | 3.008 | 3.269 |  | 1.002 | 0.940 | 1.068 |
|  | 80-89 | 3.230 | 3.079 | 3.390 |  | 3.285 | 3.136 | 3.440 |  | 1.017 | 0.951 | 1.087 |
|  | 90 and over | 1.601 | 1.513 | 1.694 |  | 2.241 | 2.002 | 2.500 |  | 1.400 | 1.234 | 1.583 |
| Gender | Male | 0.934 | 0.911 | 0.958 |  | 1.080 | 1.052 | 1.109 |  | 1.156 | 1.115 | 1.199 |
| Language | NL | 3.196 | 3.105 | 3.290 |  | 3.055 | 2.943 | 3.172 |  | 0.956 | 0.912 | 1.002 |
|  |  | Null deviance: 74605.7 on 2239 degrees of freedom | | |  | Null deviance: 73668.5 on 2239 degrees of freedom | | |  | Null deviance: 148448 on 4479 degrees of freedom | | |
|  |  | Residual deviance: 7348.7 on 2217 degrees of freedom | | |  | Residual deviance: 4908.2 on 2217 degrees of freedom | | |  | Residual deviance: 12257 on 4434 degrees of freedom | | |
|  |  | AIC: 12544 | | |  | AIC: 9654 | | |  | AIC: 22198 | | |

Effects

## Supplementary file S4.3. Interaction between year and age group

|  |  | Unweighted | | |  | Weighted | | |  | Difference | | |
| --- | --- | --- | --- | --- | --- | --- | --- | --- | --- | --- | --- | --- |
|  |  | IRR | 95%CI | |  | IRR | 95%CI | |  | IRR | 95%CI | |
|  |  |  | 2.5 % | 97.5 % |  |  | 2.5 % | 97.5 % |  |  | 2.5 % | 97.5 % |
| (Intercept) | | 4.117 | 3.724 | 4.544 |  | 2.776 | 2.539 | 3.032 |  |  |  |  |
| Year | | 1.015 | 1.000 | 1.030 |  | 1.006 | 0.994 | 1.018 |  | 0.991 | 0.973 | 1.010 |
| Age group | 15-29 | 0.044 | 0.027 | 0.068 |  | 0.043 | 0.031 | 0.060 |  | 0.996 | 0.570 | 1.772 |
|  | 30-39 | 0.092 | 0.068 | 0.124 |  | 0.091 | 0.070 | 0.116 |  | 0.982 | 0.661 | 1.465 |
|  | 40-49 | 0.315 | 0.261 | 0.380 |  | 0.322 | 0.276 | 0.374 |  | 1.021 | 0.802 | 1.302 |
|  | 60-69 | 1.962 | 1.756 | 2.195 |  | 1.901 | 1.735 | 2.083 |  | 0.969 | 0.838 | 1.119 |
|  | 70-79 | 2.299 | 2.065 | 2.563 |  | 2.276 | 2.073 | 2.501 |  | 0.990 | 0.858 | 1.142 |
|  | 80-89 | 2.475 | 2.224 | 2.756 |  | 2.483 | 2.238 | 2.754 |  | 1.003 | 0.864 | 1.164 |
|  | 90 and over | 0.875 | 0.767 | 0.999 |  | 1.240 | 0.923 | 1.644 |  | 1.416 | 1.025 | 1.935 |
| Year * Age group | 15-29 | 0.986 | 0.916 | 1.063 |  | 0.992 | 0.941 | 1.047 |  | 1.006 | 0.918 | 1.102 |
|  | 30-39 | 1.029 | 0.982 | 1.078 |  | 1.035 | 0.995 | 1.077 |  | 1.006 | 0.946 | 1.069 |
|  | 40-49 | 0.997 | 0.967 | 1.027 |  | 0.993 | 0.969 | 1.018 |  | 0.996 | 0.958 | 1.036 |
|  | 60-69 | 1.028 | 1.010 | 1.047 |  | 1.034 | 1.020 | 1.049 |  | 1.006 | 0.983 | 1.029 |
|  | 70-79 | 1.054 | 1.037 | 1.072 |  | 1.057 | 1.041 | 1.072 |  | 1.002 | 0.980 | 1.025 |
|  | 80-89 | 1.047 | 1.029 | 1.065 |  | 1.050 | 1.033 | 1.067 |  | 1.003 | 0.980 | 1.027 |
|  | 90 and over | 1.105 | 1.084 | 1.127 |  | 1.102 | 1.058 | 1.148 |  | 0.997 | 0.953 | 1.043 |
| Reason | Dementia | 0.057 | 0.050 | 0.065 |  | 0.087 | 0.075 | 0.100 |  | 1.517 | 1.254 | 1.834 |
|  | NSD | 0.524 | 0.498 | 0.551 |  | 0.822 | 0.775 | 0.871 |  | 1.568 | 1.451 | 1.694 |
|  | Others | 0.071 | 0.063 | 0.080 |  | 0.115 | 0.101 | 0.130 |  | 1.614 | 1.362 | 1.912 |
|  | Psychiatric disorders | 0.075 | 0.067 | 0.084 |  | 0.181 | 0.163 | 0.200 |  | 2.418 | 2.077 | 2.817 |
|  | Specific diseases | 0.521 | 0.495 | 0.549 |  | 0.643 | 0.604 | 0.685 |  | 1.234 | 1.138 | 1.339 |
|  | Cancers | 3.760 | 3.635 | 3.891 |  | 6.152 | 5.897 | 6.421 |  | 1.636 | 1.549 | 1.728 |
| Gender | Male | 0.934 | 0.911 | 0.958 |  | 1.079 | 1.051 | 1.108 |  | 1.155 | 1.114 | 1.198 |
| Language | NL | 3.196 | 3.105 | 3.290 |  | 3.055 | 2.943 | 3.173 |  | 0.956 | 0.912 | 1.003 |
|  |  | Null deviance: 74605.7 on 2239 degrees of freedom | | |  | Null deviance: 73669 on 2239 degrees of freedom | | |  | Null deviance: 148448 on 4479 degrees of freedom | | |
|  |  | Residual deviance: 7537.6 on 2216 degrees of freedom | | |  | Residual deviance: 5039 on 2216 degrees of freedom | | |  | Residual deviance: 12577 on 4432 degrees of freedom | | |
|  |  | AIC: 12735 | | |  | AIC: 9786.8 | | |  | AIC: 22522 | | |

Effects

## Supplementary file S4.4. Interaction between year and gender

|  |  | Unweighted | | |  | Weighted | | |  | Difference | | |
| --- | --- | --- | --- | --- | --- | --- | --- | --- | --- | --- | --- | --- |
|  |  | IRR | 95%CI | |  | IRR | 95%CI | |  | IRR | 95%CI | |
|  |  |  | 2.5 % | 97.5 % |  |  | 2.5 % | 97.5 % |  |  | 2.5 % | 97.5 % |
| (Intercept) | | 3.115 | 2.912 | 3.331 |  | 2.196 | 2.043 | 2.359 |  |  |  |  |
| Year | | 1.065 | 1.058 | 1.072 |  | 1.048 | 1.041 | 1.055 |  | 0.984 | 0.976 | 0.993 |
| Gender | Male | 0.977 | 0.922 | 1.035 |  | 1.149 | 1.081 | 1.221 |  | 1.176 | 1.082 | 1.280 |
| Year * Gender | Male | 0.993 | 0.984 | 1.001 |  | 0.990 | 0.980 | 0.999 |  | 0.997 | 0.984 | 1.010 |
| Age group | 15-29 | 0.040 | 0.032 | 0.050 |  | 0.042 | 0.035 | 0.048 |  | 1.029 | 0.794 | 1.343 |
|  | 30-39 | 0.109 | 0.095 | 0.124 |  | 0.110 | 0.098 | 0.123 |  | 1.013 | 0.850 | 1.207 |
|  | 40-49 | 0.310 | 0.284 | 0.338 |  | 0.310 | 0.289 | 0.333 |  | 1.002 | 0.896 | 1.121 |
|  | 60-69 | 2.304 | 2.191 | 2.423 |  | 2.300 | 2.207 | 2.398 |  | 0.998 | 0.936 | 1.066 |
|  | 70-79 | 3.130 | 2.983 | 3.285 |  | 3.136 | 3.008 | 3.270 |  | 1.002 | 0.940 | 1.068 |
|  | 80-89 | 3.230 | 3.079 | 3.390 |  | 3.286 | 3.137 | 3.441 |  | 1.017 | 0.952 | 1.087 |
|  | 90 and over | 1.601 | 1.513 | 1.694 |  | 2.248 | 2.008 | 2.508 |  | 1.404 | 1.238 | 1.589 |
| Reason | Dementia | 0.057 | 0.050 | 0.065 |  | 0.087 | 0.075 | 0.100 |  | 1.517 | 1.254 | 1.834 |
|  | NSD | 0.524 | 0.498 | 0.551 |  | 0.822 | 0.775 | 0.871 |  | 1.568 | 1.451 | 1.694 |
|  | Others | 0.071 | 0.063 | 0.080 |  | 0.115 | 0.101 | 0.130 |  | 1.614 | 1.362 | 1.912 |
|  | Psychiatric disorders | 0.075 | 0.067 | 0.084 |  | 0.181 | 0.163 | 0.200 |  | 2.418 | 2.077 | 2.817 |
|  | Specific diseases | 0.521 | 0.495 | 0.549 |  | 0.643 | 0.604 | 0.685 |  | 1.234 | 1.138 | 1.339 |
|  | Cancers | 3.760 | 3.635 | 3.891 |  | 6.152 | 5.897 | 6.422 |  | 1.636 | 1.549 | 1.728 |
| Language | NL | 3.196 | 3.105 | 3.290 |  | 3.055 | 2.943 | 3.172 |  | 0.956 | 0.912 | 1.002 |
|  |  | Null deviance: 74605.7 on 2239 degrees of freedom | | |  | Null deviance: 73668.5 on 2239 degrees of freedom | | |  | Null deviance: 148448 on 4479 degrees of freedom | | |
|  |  | Residual deviance: 7667.9 on 2222 degrees of freedom | | |  | Residual deviance: 5121.9 on 2222 degrees of freedom | | |  | Residual deviance: 12790 on 4444 degrees of freedom | | |
|  |  | AIC: 12854 | | |  | AIC: 9857.7 | | |  | AIC: 22711 | | |

Effects

## Supplementary file S4.5. Interaction between year and reason/language

|  |  | Unweighted | | |  | Weighted | | |  | Difference | | |
| --- | --- | --- | --- | --- | --- | --- | --- | --- | --- | --- | --- | --- |
|  |  | IRR | 95%CI | |  | IRR | 95%CI | |  | IRR | 95%CI | |
|  |  |  | 2.5 % | 97.5 % |  |  | 2.5 % | 97.5 % |  |  | 2.5 % | 97.5 % |
| (Intercept) | | 2.404 | 2.219 | 2.604 |  | 1.658 | 1.501 | 1.829 |  |  |  |  |
| Year | | 1.110 | 1.101 | 1.120 |  | 1.097 | 1.084 | 1.111 |  | 0.988 | 0.973 | 1.004 |
| Language | NL | 4.613 | 4.301 | 4.951 |  | 4.386 | 4.005 | 4.808 |  | 0.951 | 0.847 | 1.067 |
| Year * Language | NL | 0.942 | 0.932 | 0.951 |  | 0.942 | 0.930 | 0.955 |  | 1.001 | 0.984 | 1.018 |
| Age group | 15-29 | 0.040 | 0.032 | 0.050 |  | 0.042 | 0.036 | 0.048 |  | 1.030 | 0.794 | 1.343 |
|  | 30-39 | 0.109 | 0.095 | 0.124 |  | 0.110 | 0.098 | 0.123 |  | 1.013 | 0.850 | 1.207 |
|  | 40-49 | 0.310 | 0.284 | 0.338 |  | 0.311 | 0.289 | 0.333 |  | 1.002 | 0.897 | 1.121 |
|  | 60-69 | 2.304 | 2.191 | 2.423 |  | 2.301 | 2.208 | 2.398 |  | 0.999 | 0.936 | 1.066 |
|  | 70-79 | 3.130 | 2.983 | 3.285 |  | 3.133 | 3.005 | 3.267 |  | 1.001 | 0.939 | 1.067 |
|  | 80-89 | 3.230 | 3.079 | 3.390 |  | 3.287 | 3.139 | 3.443 |  | 1.018 | 0.952 | 1.088 |
|  | 90 and over | 1.601 | 1.513 | 1.694 |  | 2.249 | 2.009 | 2.509 |  | 1.405 | 1.239 | 1.589 |
| Reason | Dementia | 0.057 | 0.050 | 0.065 |  | 0.087 | 0.075 | 0.100 |  | 1.517 | 1.254 | 1.834 |
|  | NSD | 0.524 | 0.498 | 0.551 |  | 0.822 | 0.775 | 0.871 |  | 1.568 | 1.451 | 1.694 |
|  | Others | 0.071 | 0.063 | 0.080 |  | 0.115 | 0.101 | 0.130 |  | 1.614 | 1.362 | 1.912 |
|  | Psychiatric disorders | 0.075 | 0.067 | 0.084 |  | 0.181 | 0.163 | 0.200 |  | 2.418 | 2.077 | 2.817 |
|  | Specific diseases | 0.521 | 0.495 | 0.549 |  | 0.643 | 0.604 | 0.685 |  | 1.234 | 1.138 | 1.339 |
|  | Cancers | 3.760 | 3.635 | 3.891 |  | 6.152 | 5.896 | 6.421 |  | 1.636 | 1.549 | 1.728 |
| Gender | Male | 0.934 | 0.911 | 0.958 |  | 1.080 | 1.052 | 1.109 |  | 1.156 | 1.115 | 1.199 |
|  |  | Null deviance: 74605.7 on 2239 degrees of freedom | | |  | Null deviance: 73668.5 on 2239 degrees of freedom | | |  | Null deviance: 148448 on 4479 degrees of freedom | | |
|  |  | Residual deviance: 7538.2 on 2222 degrees of freedom | | |  | Residual deviance: 5049.3 on 2222 degrees of freedom | | |  | Residual deviance: 12587 on 4444 degrees of freedom | | |
|  |  | AIC: 12724 | | |  | AIC: 9785.1 | | |  | AIC: 22509 | | |

Effects

# Sensitivity file S5. Counter factual analysis

## Supplementary file S6.1. Main model (no interaction)

|  |  | Full weight | | |  | Baseline weight | | |  | Difference | | |
| --- | --- | --- | --- | --- | --- | --- | --- | --- | --- | --- | --- | --- |
|  |  | IRR | 95%CI | |  | IRR | 95%CI | |  | IRR | 95%CI | |
|  |  |  | 0.025 | 0.975 |  |  | 0.025 | 0.975 |  |  | 0.025 | 0.975 |
| (Intercept) | | 2.252 | 2.105 | 2.408 |  | 2.235 | 2.086 | 2.392 |  |  |  |  |
| Year | | 1.044 | 1.039 | 1.049 |  | 1.044 | 1.039 | 1.049 |  | 0.999 | 0.993 | 1.006 |
| Age group | 15-29 | 0.041 | 0.035 | 0.048 |  | 0.041 | 0.035 | 0.048 |  | 1.000 | 0.802 | 1.248 |
|  | 30-39 | 0.109 | 0.097 | 0.122 |  | 0.109 | 0.097 | 0.122 |  | 1.001 | 0.851 | 1.177 |
|  | 40-49 | 0.311 | 0.289 | 0.334 |  | 0.309 | 0.288 | 0.332 |  | 0.996 | 0.900 | 1.103 |
|  | 60-69 | 2.300 | 2.204 | 2.400 |  | 2.304 | 2.207 | 2.405 |  | 1.002 | 0.943 | 1.064 |
|  | 70-79 | 3.132 | 3.001 | 3.271 |  | 3.121 | 2.988 | 3.261 |  | 0.996 | 0.937 | 1.059 |
|  | 80-89 | 3.281 | 3.128 | 3.441 |  | 3.296 | 3.142 | 3.458 |  | 1.005 | 0.939 | 1.075 |
|  | 90 and over | 2.203 | 1.962 | 2.465 |  | 2.206 | 1.939 | 2.500 |  | 1.002 | 0.844 | 1.188 |
| Gender | Male | 1.076 | 1.046 | 1.105 |  | 1.075 | 1.045 | 1.106 |  | 1.000 | 0.961 | 1.039 |
| Language | NL | 3.058 | 2.949 | 3.171 |  | 3.058 | 2.948 | 3.173 |  | 1.000 | 0.950 | 1.053 |
| Reason | Dementia | 0.086 | 0.074 | 0.099 |  | 0.086 | 0.074 | 0.099 |  | 0.998 | 0.811 | 1.228 |
|  | NSD | 0.834 | 0.785 | 0.886 |  | 0.844 | 0.794 | 0.898 |  | 1.012 | 0.929 | 1.103 |
|  | Others | 0.116 | 0.102 | 0.132 |  | 0.120 | 0.105 | 0.136 |  | 1.031 | 0.861 | 1.234 |
|  | Psychiatric disorders | 0.178 | 0.160 | 0.197 |  | 0.187 | 0.168 | 0.208 |  | 1.055 | 0.909 | 1.224 |
|  | Specific diseases | 0.644 | 0.604 | 0.688 |  | 0.649 | 0.607 | 0.694 |  | 1.008 | 0.918 | 1.106 |
|  | Cancers | 6.129 | 5.867 | 6.407 |  | 6.194 | 5.922 | 6.481 |  | 1.011 | 0.949 | 1.076 |
|  |  | Null deviance: 70353.7 on 2239 degrees of freedom | | |  | Null deviance: 68224.3 on 2239 degrees of freedom | | |  | Null deviance: 138593 on 4479 degrees of freedom | | |
|  |  | Residual deviance: 4931.5 on 2223 degrees of freedom | | |  | Residual deviance: 4824.5 on 2223 degrees of freedom | | |  | Residual deviance: 9756 on 4446 degrees of freedom | | |
|  |  | AIC: 9490.1 | | |  | AIC: 9313.8 | | |  | AIC: 18804 | | |

Effects

## Supplementary file S6.2. Interaction between year and reason for euthanasia

|  |  | Full weight | | |  | Baseline weight | | |  | Difference | | |
| --- | --- | --- | --- | --- | --- | --- | --- | --- | --- | --- | --- | --- |
|  |  | IRR | 95%CI | |  | IRR | 95%CI | |  | IRR | 95%CI | |
|  |  |  | 2.5 % | 97.5 % |  |  | 2.5 % | 97.5 % |  |  | 2.5 % | 97.5 % |
| (Intercept) | | 1.386 | 1.235 | 1.553 |  | 1.375 | 1.223 | 1.542 |  |  |  |  |
| Year | | 1.129 | 1.113 | 1.146 |  | 1.129 | 1.112 | 1.147 |  | 1.000 | 0.979 | 1.022 |
| Reason | Dementia | 0.077 | 0.052 | 0.111 |  | 0.077 | 0.052 | 0.112 |  | 1.008 | 0.584 | 1.736 |
|  | NSD | 1.047 | 0.901 | 1.218 |  | 1.059 | 0.909 | 1.233 |  | 1.011 | 0.816 | 1.252 |
|  | Others | 0.232 | 0.174 | 0.306 |  | 0.233 | 0.174 | 0.309 |  | 1.005 | 0.672 | 1.503 |
|  | Psychiatric disorders | 0.471 | 0.374 | 0.589 |  | 0.474 | 0.377 | 0.593 |  | 1.008 | 0.732 | 1.389 |
|  | Specific diseases | 1.196 | 1.024 | 1.395 |  | 1.210 | 1.034 | 1.414 |  | 1.012 | 0.812 | 1.260 |
|  | Cancers | 10.800 | 9.669 | 12.084 |  | 10.925 | 9.768 | 12.242 |  | 1.012 | 0.863 | 1.186 |
| Year * Reason | Dementia | 1.018 | 0.966 | 1.074 |  | 1.017 | 0.964 | 1.074 |  | 0.999 | 0.926 | 1.077 |
|  | NSD | 0.965 | 0.944 | 0.986 |  | 0.965 | 0.944 | 0.986 |  | 1.000 | 0.970 | 1.031 |
|  | Others | 0.893 | 0.854 | 0.933 |  | 0.896 | 0.856 | 0.937 |  | 1.003 | 0.942 | 1.068 |
|  | Psychiatric disorders | 0.847 | 0.816 | 0.879 |  | 0.853 | 0.822 | 0.885 |  | 1.007 | 0.956 | 1.061 |
|  | Specific diseases | 0.904 | 0.884 | 0.925 |  | 0.903 | 0.882 | 0.924 |  | 0.998 | 0.966 | 1.032 |
|  | Cancers | 0.912 | 0.898 | 0.927 |  | 0.911 | 0.897 | 0.926 |  | 0.999 | 0.976 | 1.022 |
| Age group | 15-29 | 0.041 | 0.035 | 0.048 |  | 0.041 | 0.035 | 0.048 |  | 1.000 | 0.802 | 1.248 |
|  | 30-39 | 0.109 | 0.097 | 0.122 |  | 0.109 | 0.097 | 0.122 |  | 1.001 | 0.851 | 1.177 |
|  | 40-49 | 0.310 | 0.289 | 0.334 |  | 0.309 | 0.288 | 0.332 |  | 0.996 | 0.900 | 1.103 |
|  | 60-69 | 2.300 | 2.204 | 2.400 |  | 2.304 | 2.207 | 2.405 |  | 1.002 | 0.943 | 1.064 |
|  | 70-79 | 3.132 | 3.001 | 3.270 |  | 3.121 | 2.988 | 3.261 |  | 0.996 | 0.937 | 1.059 |
|  | 80-89 | 3.281 | 3.128 | 3.442 |  | 3.296 | 3.142 | 3.458 |  | 1.005 | 0.939 | 1.075 |
|  | 90 and over | 2.197 | 1.956 | 2.458 |  | 2.206 | 1.939 | 2.500 |  | 1.004 | 0.846 | 1.191 |
| Gender | Male | 1.076 | 1.047 | 1.106 |  | 1.075 | 1.045 | 1.106 |  | 0.999 | 0.961 | 1.039 |
| Language | NL | 3.058 | 2.949 | 3.171 |  | 3.058 | 2.948 | 3.173 |  | 1.000 | 0.950 | 1.053 |
|  |  | Null deviance: 70353.7 on 2239 degrees of freedom | | |  | Null deviance: 68224.3 on 2239 degrees of freedom | | |  | Null deviance: 138593.2 on 4479 degrees of freedom | | |
|  |  | Residual deviance: 4723.1 on 2217 degrees of freedom | | |  | Residual deviance: 4623.3 on 2217 degrees of freedom | | |  | Residual deviance: 9346.4 on 4434 degrees of freedom | | |
|  |  | AIC: 9293.6 | | |  | AIC: 9124.7 | | |  | AIC: 18418 | | |

Effects

## Supplementary file S6.3. Interaction between year and age group

|  |  | Full weight | | |  | Baseline weight | | |  | Difference | | |
| --- | --- | --- | --- | --- | --- | --- | --- | --- | --- | --- | --- | --- |
|  |  | IRR | 95%CI | |  | IRR | 95%CI | |  | IRR | 95%CI | |
|  |  |  | 2.5 % | 97.5 % |  |  | 2.5 % | 97.5 % |  |  | 2.5 % | 97.5 % |
| (Intercept) | | 2.746 | 2.507 | 3.006 |  | 2.717 | 2.478 | 2.976 |  | 1.000 | 0.983 | 1.017 |
| Year | | 1.009 | 0.997 | 1.021 |  | 1.009 | 0.997 | 1.021 |  | 1.002 | 0.622 | 1.615 |
| Age group | 15-29 | 0.043 | 0.030 | 0.060 |  | 0.043 | 0.030 | 0.060 |  | 1.000 | 0.692 | 1.446 |
|  | 30-39 | 0.091 | 0.069 | 0.117 |  | 0.091 | 0.070 | 0.117 |  | 1.002 | 0.805 | 1.248 |
|  | 40-49 | 0.318 | 0.272 | 0.371 |  | 0.318 | 0.273 | 0.371 |  | 1.000 | 0.874 | 1.143 |
|  | 60-69 | 1.919 | 1.746 | 2.110 |  | 1.918 | 1.744 | 2.110 |  | 1.004 | 0.874 | 1.152 |
|  | 70-79 | 2.286 | 2.075 | 2.519 |  | 2.294 | 2.081 | 2.529 |  | 0.996 | 0.855 | 1.159 |
|  | 80-89 | 2.497 | 2.243 | 2.781 |  | 2.487 | 2.233 | 2.770 |  | 1.003 | 0.650 | 1.543 |
|  | 90 and over | 1.213 | 0.896 | 1.622 |  | 1.217 | 0.882 | 1.651 |  | 1.000 | 0.926 | 1.080 |
| Year * Age group | 15-29 | 0.991 | 0.938 | 1.047 |  | 0.991 | 0.939 | 1.046 |  | 1.000 | 0.945 | 1.058 |
|  | 30-39 | 1.034 | 0.993 | 1.076 |  | 1.033 | 0.993 | 1.076 |  | 0.999 | 0.965 | 1.035 |
|  | 40-49 | 0.995 | 0.971 | 1.021 |  | 0.995 | 0.971 | 1.020 |  | 1.000 | 0.979 | 1.021 |
|  | 60-69 | 1.033 | 1.017 | 1.048 |  | 1.033 | 1.017 | 1.048 |  | 0.999 | 0.978 | 1.021 |
|  | 70-79 | 1.056 | 1.040 | 1.072 |  | 1.055 | 1.039 | 1.071 |  | 1.001 | 0.978 | 1.025 |
|  | 80-89 | 1.049 | 1.031 | 1.066 |  | 1.050 | 1.033 | 1.068 |  | 1.003 | 0.943 | 1.066 |
|  | 90 and over | 1.102 | 1.057 | 1.149 |  | 1.105 | 1.056 | 1.156 |  | 1.003 | 0.650 | 1.543 |
| Reason | Dementia | 0.086 | 0.074 | 0.099 |  | 0.086 | 0.074 | 0.099 |  | 0.998 | 0.811 | 1.228 |
|  | NSD | 0.834 | 0.785 | 0.886 |  | 0.844 | 0.794 | 0.898 |  | 1.012 | 0.929 | 1.103 |
|  | Others | 0.116 | 0.102 | 0.132 |  | 0.120 | 0.105 | 0.136 |  | 1.031 | 0.861 | 1.234 |
|  | Psychiatric disorders | 0.178 | 0.160 | 0.197 |  | 0.187 | 0.168 | 0.208 |  | 1.055 | 0.909 | 1.224 |
|  | Specific diseases | 0.644 | 0.604 | 0.688 |  | 0.649 | 0.607 | 0.694 |  | 1.008 | 0.918 | 1.106 |
|  | Cancers | 6.129 | 5.867 | 6.407 |  | 6.194 | 5.922 | 6.481 |  | 1.011 | 0.949 | 1.076 |
| Gender | Male | 1.075 | 1.046 | 1.105 |  | 1.075 | 1.045 | 1.106 |  | 1.000 | 0.962 | 1.040 |
| Language | NL | 3.057 | 2.949 | 3.171 |  | 3.058 | 2.948 | 3.173 |  | 1.000 | 0.950 | 1.053 |
|  |  | Null deviance: 70353.7 on 2239 degrees of freedom | | |  | Null deviance: 68224.3 on 2239 degrees of freedom | | |  | Null deviance: 138593.2 on 4479 degrees of freedom | | |
|  |  | Residual deviance: 4852.7 on 2216 degrees of freedom | | |  | Residual deviance: 4746.9 on 2216 degrees of freedom | | |  | Residual deviance: 9599.6 on 4432 degrees of freedom | | |
|  |  | AIC: 9425.2 | | |  | AIC: 9250.3 | | |  | AIC: 18675 | | |

Effects

## Supplementary file S6.4. Interaction between year and gender

|  |  | Full weight | | |  | Baseline weight | | |  | Difference | | |
| --- | --- | --- | --- | --- | --- | --- | --- | --- | --- | --- | --- | --- |
|  |  | IRR | 95%CI | |  | IRR | 95%CI | |  | IRR | 95%CI | |
|  |  |  | 2.5 % | 97.5 % |  |  | 2.5 % | 97.5 % |  |  | 2.5 % | 97.5 % |
| (Intercept) | | 2.186 | 2.032 | 2.351 |  | 2.165 | 2.010 | 2.331 |  |  |  |  |
| Year | | 1.050 | 1.043 | 1.057 |  | 1.049 | 1.042 | 1.057 |  | 1.000 | 0.990 | 1.009 |
| Gender | Male | 1.142 | 1.073 | 1.217 |  | 1.148 | 1.077 | 1.223 |  | 1.005 | 0.919 | 1.099 |
| Year * Gender | Male | 0.990 | 0.980 | 0.999 |  | 0.989 | 0.979 | 0.999 |  | 0.999 | 0.985 | 1.013 |
| Age group | 15-29 | 0.041 | 0.035 | 0.048 |  | 0.041 | 0.035 | 0.048 |  | 1.000 | 0.802 | 1.248 |
|  | 30-39 | 0.109 | 0.097 | 0.122 |  | 0.109 | 0.097 | 0.122 |  | 1.001 | 0.851 | 1.177 |
|  | 40-49 | 0.310 | 0.289 | 0.334 |  | 0.309 | 0.288 | 0.332 |  | 0.996 | 0.900 | 1.103 |
|  | 60-69 | 2.300 | 2.204 | 2.400 |  | 2.304 | 2.207 | 2.405 |  | 1.002 | 0.943 | 1.064 |
|  | 70-79 | 3.133 | 3.001 | 3.271 |  | 3.121 | 2.988 | 3.261 |  | 0.996 | 0.937 | 1.059 |
|  | 80-89 | 3.282 | 3.129 | 3.443 |  | 3.296 | 3.142 | 3.458 |  | 1.004 | 0.939 | 1.075 |
|  | 90 and over | 2.204 | 1.962 | 2.466 |  | 2.206 | 1.939 | 2.500 |  | 1.001 | 0.844 | 1.187 |
| Reason | Dementia | 0.086 | 0.074 | 0.099 |  | 0.086 | 0.074 | 0.099 |  | 0.998 | 0.811 | 1.228 |
|  | NSD | 0.834 | 0.785 | 0.886 |  | 0.844 | 0.794 | 0.898 |  | 1.012 | 0.929 | 1.104 |
|  | Others | 0.116 | 0.102 | 0.132 |  | 0.120 | 0.105 | 0.136 |  | 1.031 | 0.861 | 1.234 |
|  | Psychiatric disorders | 0.178 | 0.160 | 0.197 |  | 0.187 | 0.168 | 0.208 |  | 1.055 | 0.909 | 1.224 |
|  | Specific diseases | 0.644 | 0.604 | 0.688 |  | 0.649 | 0.607 | 0.694 |  | 1.008 | 0.918 | 1.106 |
|  | Cancers | 6.129 | 5.867 | 6.407 |  | 6.194 | 5.922 | 6.481 |  | 1.010 | 0.949 | 1.076 |
| Language | NL | 3.058 | 2.949 | 3.171 |  | 3.058 | 2.948 | 3.173 |  | 1.000 | 0.950 | 1.053 |
|  |  | Null deviance: 70353.7 on 2239 degrees of freedom | | |  | Null deviance: 68224.3 on 2239 degrees of freedom | | |  | Null deviance: 138593.2 on 4479 degrees of freedom | | |
|  |  | Residual deviance: 4927.2 on 2222 degrees of freedom | | |  | Residual deviance: 4819.4 on 2222 degrees of freedom | | |  | Residual deviance: 9746.6 on 4444 degrees of freedom | | |
|  |  | AIC: 9487.7 | | |  | AIC: 9310.8 | | |  | AIC: 18798 | | |

Effects

## Supplementary file S6.5. Interaction between year and reason/language

|  |  | Full weight | | |  | Baseline weight | | |  | Difference | | |
| --- | --- | --- | --- | --- | --- | --- | --- | --- | --- | --- | --- | --- |
|  |  | IRR | 95%CI | |  | IRR | 95%CI | |  | IRR | 95%CI | |
|  |  |  | 2.5 % | 97.5 % |  |  | 2.5 % | 97.5 % |  |  | 2.5 % | 97.5 % |
| (Intercept) | | 1.663 | 1.510 | 1.830 |  | 1.651 | 1.498 | 1.819 |  |  |  |  |
| Year | | 1.098 | 1.085 | 1.111 |  | 1.097 | 1.084 | 1.110 |  | 1.000 | 0.983 | 1.017 |
| Language | NL | 4.391 | 4.022 | 4.798 |  | 4.392 | 4.022 | 4.802 |  | 1.000 | 0.883 | 1.134 |
| Year * Language | NL | 0.942 | 0.930 | 0.954 |  | 0.942 | 0.929 | 0.954 |  | 0.999 | 0.981 | 1.018 |
| Age group | 15-29 | 0.041 | 0.035 | 0.048 |  | 0.041 | 0.035 | 0.048 |  | 1.000 | 0.801 | 1.247 |
|  | 30-39 | 0.109 | 0.097 | 0.122 |  | 0.109 | 0.097 | 0.122 |  | 1.000 | 0.850 | 1.177 |
|  | 40-49 | 0.311 | 0.289 | 0.334 |  | 0.309 | 0.288 | 0.332 |  | 0.996 | 0.900 | 1.103 |
|  | 60-69 | 2.301 | 2.205 | 2.401 |  | 2.304 | 2.207 | 2.405 |  | 1.001 | 0.942 | 1.064 |
|  | 70-79 | 3.131 | 2.999 | 3.269 |  | 3.121 | 2.988 | 3.261 |  | 0.997 | 0.938 | 1.060 |
|  | 80-89 | 3.285 | 3.132 | 3.445 |  | 3.296 | 3.142 | 3.458 |  | 1.004 | 0.938 | 1.074 |
|  | 90 and over | 2.206 | 1.964 | 2.468 |  | 2.206 | 1.939 | 2.500 |  | 1.000 | 0.843 | 1.186 |
| Reason | Dementia | 0.086 | 0.074 | 0.099 |  | 0.086 | 0.074 | 0.099 |  | 0.998 | 0.811 | 1.228 |
|  | NSD | 0.834 | 0.785 | 0.886 |  | 0.844 | 0.794 | 0.898 |  | 1.012 | 0.929 | 1.103 |
|  | Others | 0.116 | 0.102 | 0.132 |  | 0.120 | 0.105 | 0.136 |  | 1.031 | 0.861 | 1.234 |
|  | Psychiatric disorders | 0.178 | 0.160 | 0.197 |  | 0.187 | 0.168 | 0.208 |  | 1.055 | 0.909 | 1.224 |
|  | Specific diseases | 0.644 | 0.604 | 0.688 |  | 0.649 | 0.607 | 0.694 |  | 1.008 | 0.918 | 1.106 |
|  | Cancers | 6.129 | 5.866 | 6.406 |  | 6.194 | 5.922 | 6.481 |  | 1.011 | 0.949 | 1.076 |
| Gender | Male | 1.076 | 1.046 | 1.105 |  | 1.075 | 1.045 | 1.106 |  | 1.000 | 0.961 | 1.039 |
|  |  | Null deviance: 70353.7 on 2239 degrees of freedom | | |  | Null deviance: 68224.3 on 2239 degrees of freedom | | |  | Null deviance: 138593.2 on 4479 degrees of freedom | | |
|  |  | Residual deviance: 4848.5 on 2222 degrees of freedom | | |  | Residual deviance: 4741.9 on 2222 degrees of freedom | | |  | Residual deviance: 9590.4 on 4444 degrees of freedom | | |
|  |  | AIC: 9409 | | |  | AIC: 9233.2 | | |  | AIC: 18642 | | |

Effects

# Sensitivity file S6. Counter factual, sensitivity check on Flanders and Wallonia populations only

## Supplementary file S6.1. Main model (no interaction)

|  |  | Full weight | | |  | Baseline weight | | |  | Difference | | |
| --- | --- | --- | --- | --- | --- | --- | --- | --- | --- | --- | --- | --- |
|  |  | IRR | 95%CI | |  | IRR | 95%CI | |  | IRR | 95%CI | |
|  |  |  | 0.025 | 0.975 |  |  | 0.025 | 0.975 |  |  | 0.025 | 0.975 |
| (Intercept) | | 2.264 | 2.118 | 2.419 |  | 2.242 | 2.095 | 2.399 |  |  |  |  |
| Year | | 1.043 | 1.038 | 1.048 |  | 1.042 | 1.037 | 1.047 |  | 0.999 | 0.993 | 1.006 |
| Age group | 15-29 | 0.042 | 0.035 | 0.048 |  | 0.041 | 0.036 | 0.048 |  | 1.000 | 0.806 | 1.240 |
|  | 30-39 | 0.110 | 0.098 | 0.123 |  | 0.110 | 0.098 | 0.123 |  | 1.001 | 0.853 | 1.174 |
|  | 40-49 | 0.311 | 0.289 | 0.333 |  | 0.309 | 0.289 | 0.331 |  | 0.996 | 0.902 | 1.100 |
|  | 60-69 | 2.300 | 2.207 | 2.397 |  | 2.304 | 2.210 | 2.402 |  | 1.002 | 0.944 | 1.062 |
|  | 70-79 | 3.136 | 3.008 | 3.269 |  | 3.123 | 2.994 | 3.258 |  | 0.996 | 0.939 | 1.057 |
|  | 80-89 | 3.285 | 3.136 | 3.440 |  | 3.299 | 3.149 | 3.456 |  | 1.004 | 0.941 | 1.072 |
|  | 90 and over | 2.247 | 2.008 | 2.507 |  | 2.257 | 1.989 | 2.550 |  | 1.004 | 0.850 | 1.186 |
| Gender | Male | 1.080 | 1.052 | 1.109 |  | 1.080 | 1.051 | 1.109 |  | 1.000 | 0.963 | 1.038 |
| Language | NL | 3.055 | 2.943 | 3.172 |  | 3.056 | 2.942 | 3.176 |  | 1.001 | 0.948 | 1.056 |
| Reason | Dementia | 0.087 | 0.075 | 0.100 |  | 0.087 | 0.075 | 0.100 |  | 0.999 | 0.818 | 1.221 |
|  | NSD | 0.822 | 0.775 | 0.871 |  | 0.834 | 0.786 | 0.886 |  | 1.015 | 0.933 | 1.104 |
|  | Others | 0.115 | 0.101 | 0.130 |  | 0.119 | 0.105 | 0.134 |  | 1.034 | 0.868 | 1.231 |
|  | Psychiatric disorders | 0.181 | 0.163 | 0.200 |  | 0.191 | 0.173 | 0.211 |  | 1.059 | 0.918 | 1.222 |
|  | Specific diseases | 0.643 | 0.604 | 0.685 |  | 0.649 | 0.609 | 0.693 |  | 1.009 | 0.922 | 1.105 |
|  | Cancers | 6.152 | 5.897 | 6.422 |  | 6.232 | 5.966 | 6.512 |  | 1.013 | 0.953 | 1.077 |
|  |  | *Null deviance: 73668.5 on 2239 degrees of freedom* | | |  | *Null deviance: 71353.8 on 2239 degrees of freedom* | | |  | *Null deviance: 145041 on 4479 degrees of freedom* | | |
|  |  | *Residual deviance: 5126.8 on 2223 degrees of freedom* | | |  | *Residual deviance: 5000.7 on 2223 degrees of freedom* | | |  | *Residual deviance: 10128 on 4446 degrees of freedom* | | |
|  |  | *AIC: 9860.6* | | |  | *AIC: 9656.8* | | |  | *AIC: 19517* | | |

Effects

## Supplementary file S6.2. Interaction between year and reason for euthanasia

|  |  | Full weight | | |  | Baseline weight | | |  | Difference | | |
| --- | --- | --- | --- | --- | --- | --- | --- | --- | --- | --- | --- | --- |
|  |  | IRR | 95%CI | |  | IRR | 95%CI | |  | IRR | 95%CI | |
|  |  |  | 2.5 % | 97.5 % |  |  | 2.5 % | 97.5 % |  |  | 2.5 % | 97.5 % |
| (Intercept) | | 1.399 | 1.250 | 1.562 |  | 1.388 | 1.238 | 1.552 |  |  |  |  |
| Year | | 1.127 | 1.111 | 1.143 |  | 1.127 | 1.110 | 1.143 |  | 1.000 | 0.980 | 1.021 |
| Reason | Dementia | 0.078 | 0.053 | 0.111 |  | 0.078 | 0.053 | 0.113 |  | 1.017 | 0.967 | 1.071 |
|  | NSD | 1.039 | 0.897 | 1.202 |  | 1.049 | 0.905 | 1.216 |  | 0.964 | 0.944 | 0.984 |
|  | Others | 0.230 | 0.173 | 0.301 |  | 0.231 | 0.174 | 0.303 |  | 0.892 | 0.855 | 0.932 |
|  | Psychiatric disorders | 0.481 | 0.386 | 0.597 |  | 0.485 | 0.389 | 0.601 |  | 0.846 | 0.816 | 0.876 |
|  | Specific diseases | 1.189 | 1.024 | 1.381 |  | 1.203 | 1.033 | 1.399 |  | 0.905 | 0.885 | 0.925 |
|  | Cancers | 10.768 | 9.676 | 12.002 |  | 10.887 | 9.769 | 12.154 |  | 0.913 | 0.899 | 0.927 |
| Year * Reason | Dementia | 1.017 | 0.967 | 1.071 |  | 1.016 | 0.965 | 1.071 |  | 1.008 | 0.597 | 1.701 |
|  | NSD | 0.964 | 0.944 | 0.984 |  | 0.964 | 0.944 | 0.985 |  | 1.010 | 0.820 | 1.244 |
|  | Others | 0.892 | 0.855 | 0.932 |  | 0.896 | 0.857 | 0.935 |  | 1.005 | 0.680 | 1.485 |
|  | Psychiatric disorders | 0.846 | 0.816 | 0.876 |  | 0.852 | 0.822 | 0.883 |  | 1.007 | 0.741 | 1.370 |
|  | Specific diseases | 0.905 | 0.885 | 0.925 |  | 0.903 | 0.883 | 0.924 |  | 1.011 | 0.817 | 1.251 |
|  | Cancers | 0.913 | 0.899 | 0.927 |  | 0.913 | 0.898 | 0.927 |  | 1.011 | 0.867 | 1.179 |
| Age group | 15-29 | 0.042 | 0.035 | 0.048 |  | 0.041 | 0.036 | 0.048 |  | 1.000 | 0.806 | 1.240 |
|  | 30-39 | 0.110 | 0.098 | 0.123 |  | 0.110 | 0.098 | 0.123 |  | 1.001 | 0.853 | 1.174 |
|  | 40-49 | 0.310 | 0.289 | 0.333 |  | 0.309 | 0.289 | 0.331 |  | 0.996 | 0.902 | 1.100 |
|  | 60-69 | 2.300 | 2.207 | 2.397 |  | 2.304 | 2.210 | 2.402 |  | 1.002 | 0.944 | 1.062 |
|  | 70-79 | 3.135 | 3.008 | 3.269 |  | 3.123 | 2.994 | 3.258 |  | 0.996 | 0.939 | 1.057 |
|  | 80-89 | 3.285 | 3.136 | 3.440 |  | 3.299 | 3.149 | 3.456 |  | 1.004 | 0.940 | 1.072 |
|  | 90 and over | 2.241 | 2.002 | 2.500 |  | 2.257 | 1.989 | 2.550 |  | 1.007 | 0.852 | 1.189 |
| Gender | Male | 1.080 | 1.052 | 1.109 |  | 1.080 | 1.051 | 1.109 |  | 1.000 | 0.963 | 1.038 |
| Language | NL | 3.055 | 2.943 | 3.172 |  | 3.056 | 2.942 | 3.176 |  | 1.001 | 0.948 | 1.056 |
|  |  | Null deviance: 73668.5 on 2239 degrees of freedom | | |  | Null deviance: 71353.8 on 2239 degrees of freedom | | |  | Null deviance: 145040.7 on 4479 degrees of freedom | | |
|  |  | Residual deviance: 4908.2 on 2217 degrees of freedom | | |  | Residual deviance: 4791.9 on 2217 degrees of freedom | | |  | Residual deviance: 9700.1 on 4434 degrees of freedom | | |
|  |  | AIC: 9654 | | |  | AIC: 9460 | | |  | AIC: 19114 | | |

Effects

## Supplementary file S6.3. Interaction between year and age group

|  |  | Full weight | | |  | Baseline weight | | |  | Difference | | |
| --- | --- | --- | --- | --- | --- | --- | --- | --- | --- | --- | --- | --- |
|  |  | IRR | 95%CI | |  | IRR | 95%CI | |  | IRR | 95%CI | |
|  |  |  | 2.5 % | 97.5 % |  |  | 2.5 % | 97.5 % |  |  | 2.5 % | 97.5 % |
| (Intercept) | | 2.776 | 2.539 | 3.032 |  | 2.738 | 2.502 | 2.993 |  |  |  |  |
| Year | | 1.006 | 0.994 | 1.018 |  | 1.006 | 0.995 | 1.018 |  | 1.000 | 0.984 | 1.017 |
| Age group | 15-29 | 0.043 | 0.031 | 0.060 |  | 0.043 | 0.031 | 0.060 |  | 1.002 | 0.630 | 1.594 |
|  | 30-39 | 0.091 | 0.070 | 0.116 |  | 0.091 | 0.070 | 0.117 |  | 1.001 | 0.698 | 1.437 |
|  | 40-49 | 0.322 | 0.276 | 0.374 |  | 0.322 | 0.277 | 0.374 |  | 1.001 | 0.810 | 1.239 |
|  | 60-69 | 1.901 | 1.735 | 2.083 |  | 1.901 | 1.734 | 2.085 |  | 1.000 | 0.878 | 1.139 |
|  | 70-79 | 2.276 | 2.073 | 2.501 |  | 2.287 | 2.081 | 2.514 |  | 1.005 | 0.879 | 1.148 |
|  | 80-89 | 2.483 | 2.238 | 2.754 |  | 2.476 | 2.230 | 2.747 |  | 0.997 | 0.861 | 1.155 |
|  | 90 and over | 1.240 | 0.923 | 1.644 |  | 1.246 | 0.911 | 1.679 |  | 1.005 | 0.659 | 1.529 |
| Year * Age group | 15-29 | 0.992 | 0.941 | 1.047 |  | 0.992 | 0.941 | 1.046 |  | 1.000 | 0.927 | 1.077 |
|  | 30-39 | 1.035 | 0.995 | 1.077 |  | 1.034 | 0.995 | 1.076 |  | 1.000 | 0.945 | 1.057 |
|  | 40-49 | 0.993 | 0.969 | 1.018 |  | 0.993 | 0.969 | 1.017 |  | 1.000 | 0.966 | 1.035 |
|  | 60-69 | 1.034 | 1.020 | 1.049 |  | 1.034 | 1.019 | 1.049 |  | 1.000 | 0.980 | 1.021 |
|  | 70-79 | 1.057 | 1.041 | 1.072 |  | 1.055 | 1.040 | 1.071 |  | 0.999 | 0.978 | 1.020 |
|  | 80-89 | 1.050 | 1.033 | 1.067 |  | 1.051 | 1.034 | 1.068 |  | 1.001 | 0.978 | 1.024 |
|  | 90 and over | 1.102 | 1.058 | 1.148 |  | 1.105 | 1.057 | 1.155 |  | 1.003 | 0.944 | 1.065 |
| Reason | Dementia | 0.087 | 0.075 | 0.100 |  | 0.087 | 0.075 | 0.100 |  | 0.999 | 0.818 | 1.221 |
|  | NSD | 0.822 | 0.775 | 0.871 |  | 0.834 | 0.786 | 0.886 |  | 1.015 | 0.933 | 1.104 |
|  | Others | 0.115 | 0.101 | 0.130 |  | 0.119 | 0.105 | 0.134 |  | 1.034 | 0.868 | 1.231 |
|  | Psychatric disorders | 0.181 | 0.163 | 0.200 |  | 0.191 | 0.173 | 0.211 |  | 1.059 | 0.918 | 1.222 |
|  | Specific diseases | 0.643 | 0.604 | 0.685 |  | 0.649 | 0.609 | 0.693 |  | 1.009 | 0.922 | 1.105 |
|  | Cancers | 6.152 | 5.897 | 6.421 |  | 6.232 | 5.966 | 6.512 |  | 1.013 | 0.953 | 1.077 |
| Gender | Male | 1.079 | 1.051 | 1.108 |  | 1.080 | 1.051 | 1.109 |  | 1.001 | 0.963 | 1.039 |
| Language | NL | 3.055 | 2.943 | 3.173 |  | 3.056 | 2.942 | 3.176 |  | 1.000 | 0.948 | 1.055 |
|  |  | Null deviance: 73669 on 2239 degrees of freedom | | |  | Null deviance: 71354 on 2239 degrees of freedom | | |  | Null deviance: 145041 on 4479 degrees of freedom | | |
|  |  | Residual deviance: 5039 on 2216 degrees of freedom | | |  | Residual deviance: 4915 on 2216 degrees of freedom | | |  | Residual deviance: 9954 on 4432 degrees of freedom | | |
|  |  | AIC: 9786.8 | | |  | AIC: 9585 | | |  | AIC: 19372 | | |

Effects

## Supplementary file S6.4. Interaction between year and gender

|  |  | Full weight | | |  | Baseline weight | | |  | Difference | | |
| --- | --- | --- | --- | --- | --- | --- | --- | --- | --- | --- | --- | --- |
|  |  | IRR | 95%CI | |  | IRR | 95%CI | |  | IRR | 95%CI | |
|  |  |  | 2.5 % | 97.5 % |  |  | 2.5 % | 97.5 % |  |  | 2.5 % | 97.5 % |
| (Intercept) | | 2.196 | 2.043 | 2.359 |  | 2.170 | 2.017 | 2.334 |  |  |  |  |
| Year | | 1.048 | 1.041 | 1.055 |  | 1.048 | 1.041 | 1.055 |  | 1.000 | 0.991 | 1.009 |
| Gender | Male | 1.149 | 1.081 | 1.221 |  | 1.154 | 1.086 | 1.227 |  | 1.005 | 0.921 | 1.095 |
| Year * Gender | Male | 0.990 | 0.980 | 0.999 |  | 0.989 | 0.979 | 0.998 |  | 0.999 | 0.986 | 1.012 |
| Age group | 15-29 | 0.042 | 0.035 | 0.048 |  | 0.041 | 0.036 | 0.048 |  | 1.000 | 0.806 | 1.240 |
|  | 30-39 | 0.110 | 0.098 | 0.123 |  | 0.110 | 0.098 | 0.123 |  | 1.001 | 0.853 | 1.174 |
|  | 40-49 | 0.310 | 0.289 | 0.333 |  | 0.309 | 0.289 | 0.331 |  | 0.996 | 0.902 | 1.100 |
|  | 60-69 | 2.300 | 2.207 | 2.398 |  | 2.304 | 2.210 | 2.402 |  | 1.002 | 0.944 | 1.062 |
|  | 70-79 | 3.136 | 3.008 | 3.270 |  | 3.123 | 2.994 | 3.258 |  | 0.996 | 0.939 | 1.057 |
|  | 80-89 | 3.286 | 3.137 | 3.441 |  | 3.299 | 3.149 | 3.456 |  | 1.004 | 0.940 | 1.072 |
|  | 90 and over | 2.248 | 2.008 | 2.508 |  | 2.257 | 1.989 | 2.550 |  | 1.004 | 0.849 | 1.185 |
| Reason | Dementia | 0.087 | 0.075 | 0.100 |  | 0.087 | 0.075 | 0.100 |  | 0.999 | 0.818 | 1.221 |
|  | NSD | 0.822 | 0.775 | 0.871 |  | 0.834 | 0.786 | 0.886 |  | 1.015 | 0.933 | 1.104 |
|  | Others | 0.115 | 0.101 | 0.130 |  | 0.119 | 0.105 | 0.134 |  | 1.034 | 0.868 | 1.231 |
|  | Psychiatric disorders | 0.181 | 0.163 | 0.200 |  | 0.191 | 0.173 | 0.211 |  | 1.059 | 0.918 | 1.222 |
|  | Specific diseases | 0.643 | 0.604 | 0.685 |  | 0.649 | 0.609 | 0.693 |  | 1.009 | 0.922 | 1.105 |
|  | Cancers | 6.152 | 5.897 | 6.422 |  | 6.232 | 5.966 | 6.512 |  | 1.013 | 0.953 | 1.077 |
| Language | NL | 3.055 | 2.943 | 3.172 |  | 3.056 | 2.942 | 3.176 |  | 1.001 | 0.948 | 1.056 |
|  |  | Null deviance: 73668.5 on 2239 degrees of freedom | | |  | Null deviance: 71353.8 on 2239 degrees of freedom | | |  | Null deviance: 145041 on 4479 degrees of freedom | | |
|  |  | Residual deviance: 5121.9 on 2222 degrees of freedom | | |  | Residual deviance: 4995.1 on 2222 degrees of freedom | | |  | Residual deviance: 10117 on 4444 degrees of freedom | | |
|  |  | AIC: 9857.7 | | |  | AIC: 9653.2 | | |  | AIC: 19511 | | |

Effects

## Supplementary file S6.5. Interaction between year and reason/language

|  |  | Full weight | | |  | Baseline weight | | |  | Difference | | |
| --- | --- | --- | --- | --- | --- | --- | --- | --- | --- | --- | --- | --- |
|  |  | IRR | 95%CI | |  | IRR | 95%CI | |  | IRR | 95%CI | |
|  |  |  | 2.5 % | 97.5 % |  |  | 2.5 % | 97.5 % |  |  | 2.5 % | 97.5 % |
| (Intercept) | | 1.658 | 1.501 | 1.829 |  | 1.643 | 1.486 | 1.815 |  |  |  |  |
| Year | | 1.097 | 1.084 | 1.111 |  | 1.097 | 1.083 | 1.111 |  | 1.000 | 0.982 | 1.017 |
| Language | NL | 4.386 | 4.005 | 4.808 |  | 4.385 | 4.002 | 4.811 |  | 1.000 | 0.878 | 1.138 |
| Year * Language | NL | 0.942 | 0.930 | 0.955 |  | 0.942 | 0.929 | 0.955 |  | 1.000 | 0.981 | 1.019 |
| Age group | 15-29 | 0.042 | 0.036 | 0.048 |  | 0.041 | 0.036 | 0.048 |  | 0.999 | 0.806 | 1.240 |
|  | 30-39 | 0.110 | 0.098 | 0.123 |  | 0.110 | 0.098 | 0.123 |  | 1.001 | 0.853 | 1.174 |
|  | 40-49 | 0.311 | 0.289 | 0.333 |  | 0.309 | 0.289 | 0.331 |  | 0.996 | 0.902 | 1.100 |
|  | 60-69 | 2.301 | 2.208 | 2.398 |  | 2.304 | 2.210 | 2.402 |  | 1.001 | 0.944 | 1.062 |
|  | 70-79 | 3.133 | 3.005 | 3.267 |  | 3.123 | 2.994 | 3.258 |  | 0.997 | 0.939 | 1.058 |
|  | 80-89 | 3.287 | 3.139 | 3.443 |  | 3.299 | 3.149 | 3.456 |  | 1.004 | 0.940 | 1.072 |
|  | 90 and over | 2.249 | 2.009 | 2.509 |  | 2.257 | 1.989 | 2.550 |  | 1.004 | 0.849 | 1.185 |
| Reason | Dementia | 0.087 | 0.075 | 0.100 |  | 0.087 | 0.075 | 0.100 |  | 0.999 | 0.818 | 1.221 |
|  | NSD | 0.822 | 0.775 | 0.871 |  | 0.834 | 0.786 | 0.886 |  | 1.015 | 0.933 | 1.104 |
|  | Others | 0.115 | 0.101 | 0.130 |  | 0.119 | 0.105 | 0.134 |  | 1.034 | 0.868 | 1.231 |
|  | Psychiatric disorders | 0.181 | 0.163 | 0.200 |  | 0.191 | 0.173 | 0.211 |  | 1.059 | 0.918 | 1.222 |
|  | Specific diseases | 0.643 | 0.604 | 0.685 |  | 0.649 | 0.609 | 0.693 |  | 1.009 | 0.922 | 1.105 |
|  | Cancers | 6.152 | 5.896 | 6.421 |  | 6.232 | 5.966 | 6.512 |  | 1.013 | 0.953 | 1.077 |
| Gender | Male | 1.080 | 1.052 | 1.109 |  | 1.080 | 1.051 | 1.109 |  | 1.000 | 0.963 | 1.038 |
|  |  | Null deviance: 73668.5 on 2239 degrees of freedom | | |  | Null deviance: 71353.8 on 2239 degrees of freedom | | |  | Null deviance: 145040.7 on 4479 degrees of freedom | | |
|  |  | Residual deviance: 5049.3 on 2222 degrees of freedom | | |  | Residual deviance: 4924.3 on 2222 degrees of freedom | | |  | Residual deviance: 9973.6 on 4444 degrees of freedom | | |
|  |  | AIC: 9785.1 | | |  | AIC: 9582.3 | | |  | AIC: 19367 | | |

Effects

# Supplementary file S7. Interaction by the reason for euthanasia and gender

|  |  | Unweighted | | |  | Weighted | | |  | Difference | | |
| --- | --- | --- | --- | --- | --- | --- | --- | --- | --- | --- | --- | --- |
|  |  | IRR | 95%CI | |  | IRR | 95%CI | |  | IRR | 95%CI | |
|  |  |  | 2.5 % | 97.5 % |  |  | 2.5 % | 97.5 % |  |  | 2.5 % | 97.5 % |
| (Intercept) | | 1.970 | 1.797 | 2.157 |  | 1.386 | 1.235 | 1.553 |  |  |  |  |
| Year | | 1.043 | 1.038 | 1.048 |  | 1.042 | 1.037 | 1.047 |  | 1.061 | 1.056 | 1.066 |
| Gender | Male | 0.791 | 0.730 | 0.857 |  | 0.777 | 0.715 | 0.845 |  | 1.143 | 1.031 | 1.267 |
| Reason | Dementia | 0.072 | 0.059 | 0.087 |  | 0.072 | 0.059 | 0.088 |  | 1.535 | 1.163 | 2.023 |
|  | NSD | 0.719 | 0.665 | 0.778 |  | 0.722 | 0.666 | 0.782 |  | 1.478 | 1.332 | 1.641 |
|  | Others | 0.113 | 0.096 | 0.132 |  | 0.116 | 0.098 | 0.135 |  | 1.591 | 1.271 | 1.991 |
|  | Psychiatric disorders | 0.208 | 0.183 | 0.235 |  | 0.217 | 0.191 | 0.245 |  | 2.361 | 1.956 | 2.854 |
|  | Specific diseases | 0.581 | 0.534 | 0.632 |  | 0.585 | 0.536 | 0.637 |  | 1.263 | 1.129 | 1.412 |
|  | Cancers | 5.131 | 4.852 | 5.430 |  | 5.170 | 4.884 | 5.477 |  | 1.674 | 1.555 | 1.802 |
| Male * Reason | Dementia | 1.517 | 1.146 | 2.007 |  | 1.507 | 1.130 | 2.010 |  | 0.960 | 0.651 | 1.416 |
|  | NSD | 1.350 | 1.200 | 1.519 |  | 1.390 | 1.231 | 1.569 |  | 1.180 | 1.007 | 1.384 |
|  | Others | 1.042 | 0.811 | 1.336 |  | 1.070 | 0.830 | 1.377 |  | 1.066 | 0.752 | 1.512 |
|  | Psychiatric disorders | 0.678 | 0.546 | 0.839 |  | 0.703 | 0.566 | 0.871 |  | 1.021 | 0.734 | 1.424 |
|  | Specific diseases | 1.261 | 1.111 | 1.433 |  | 1.277 | 1.121 | 1.456 |  | 0.956 | 0.809 | 1.130 |
|  | Cancers | 1.484 | 1.361 | 1.619 |  | 1.512 | 1.383 | 1.653 |  | 0.958 | 0.856 | 1.073 |
| Age group | 15-29 | 0.042 | 0.035 | 0.048 |  | 0.041 | 0.036 | 0.048 |  | 1.015 | 0.781 | 1.327 |
|  | 30-39 | 0.110 | 0.098 | 0.123 |  | 0.110 | 0.098 | 0.123 |  | 1.007 | 0.844 | 1.202 |
|  | 40-49 | 0.311 | 0.289 | 0.333 |  | 0.309 | 0.289 | 0.331 |  | 1.002 | 0.896 | 1.122 |
|  | 60-69 | 2.300 | 2.207 | 2.398 |  | 2.304 | 2.210 | 2.402 |  | 0.998 | 0.934 | 1.066 |
|  | 70-79 | 3.136 | 3.008 | 3.270 |  | 3.123 | 2.995 | 3.259 |  | 1.001 | 0.938 | 1.067 |
|  | 80-89 | 3.285 | 3.136 | 3.441 |  | 3.299 | 3.149 | 3.456 |  | 1.016 | 0.949 | 1.087 |
|  | 90 and over | 2.190 | 1.956 | 2.444 |  | 2.199 | 1.937 | 2.485 |  | 1.376 | 1.210 | 1.562 |
| Language | NL | 3.053 | 2.941 | 3.170 |  | 3.055 | 2.941 | 3.174 |  | 0.960 | 0.917 | 1.006 |
|  |  | Null deviance: 74605.7 on 2239 degrees of freedom | | |  | Null deviance: 70353.7 on 2239 degrees of freedom | | |  | Null deviance: 145353 on 4479 degrees of freedom | | |
|  |  | Residual deviance: 7446.6 on 2217 degrees of freedom | | |  | Residual deviance: 4799.2 on 2217 degrees of freedom | | |  | Residual deviance: 12246 on 4434 degrees of freedom | | |
|  |  | AIC: 12642 | | |  | AIC: 9369.7 | | |  | AIC: 22012 | | |

Effects
